# Stabilization of charged domains in ferroelectric BiFeO_3_ nanoneedles

**DOI:** 10.1038/s41467-026-76259-z

**Published:** 2026-08-26

**Authors:** Francisco Guzman, Christopher Addiego, Aiden Ross, Moaz Waqar, Long-Qing Chen, Xiaoqing Pan

**Affiliations:** 1 Department of Materials Science and Engineering, University of California, Irvine, CA 92697 USA; 2 Department of Physics and Astronomy, University of California, Irvine, CA 92697 USA; 3 Materials Science and Engineering Department, Pennsylvania State University, University Park, PA 16802 USA; 4 Irvine Materials Research Institute, University of California, Irvine, CA 92697 USA

**Keywords:** Nanoscale materials, Theory and computation, Information storage

## Abstract

Stabilizing ferroelectric domain configurations is critical to driving the exploration of the fundamental physics and governing principles of domain formation in ferroelectrics. Charged domain walls have attracted interest in nanoelectronics due to their high conductivity but are usually not utilized due to their conventional instability. In this study, we investigate charged domains impacted by reduced dimensionality and surface boundary conditions in BiFeO_3_ nanoneedles. The BiFeO_3_ nanoneedle’s domain structure was examined in three dimensions using a low-order tilt projection series collected by scanning transmission electron microscopy. We find that surfaces of varying surface curvature can facilitate the formation of charge-compensating surface terminations and can play a role in the stabilization of charged domains in BiFeO_3_. Additionally, phase-field simulations support the formation of charged domains in the bulk of BiFeO_3_ nanoneedles by charge domains forming to reduce the total energy at the needle interior through domain wall reconstruction. Our results pave a novel pathway to stabilize inherently unstable charged domains in ferroelectric oxides and provide insights into geometric boundary condition engineering of thin films and nanostructures.

## Abstract (web summary)

The authors propose a top down strategy of modifying the surface boundary conditions in a BiFeO_3_ nanoneedle sample through ion milling. Surface curvature gradients and terminations induce charge compensation and ferroeleastic effects that facilitate charged domain wall stabilization.

## Introduction

Interest in domain walls in nanoelectronics prompted further study of charged domain walls (CDWs) due to their higher DC conductivity than neutral domain walls^1–4^. As a result, extensive methods have been developed to control the stability of inherently unstable CDWs in BiFeO_3_ (BFO) via DC electric field poling, varying temperatures, and defect engineering^5,6^. Additionally, geometrical confinement like that found in nanoislands and nanodot nanostructures can increase stability toward the formation of CDWs in BFO^7,8^. The evolution of nanostructures in BFO has seen considerable progress over the years. Advancements in BFO nanostructures aim to enhance BFO performance for applications in electronics, optoelectronics, and spintronics, among others. One implication of nanostructures is the surface shape effects not found in planar thin films. Recent work has shown that surface curvature can tune the polar states of ferroelectric domains in LiNbO_3_ bulk crystals^9^, control the stability of topological polar states in PbZr_0.52_Ti_0.48_O_3_ nanotubes^10^, and generate geometry-governed nonlocal chiral symmetry-breaking effects in thin magnetic curved shells^7,11,12^. However, compared to studies on curvilinear magnetism, curvature effects in ferroelectric materials are underdeveloped. Exploration of the effects of surface boundary conditions on the domain wall configuration in ferroelectrics could assist the stabilization of exotic polarization topological textures and unstable domain configurations like CDWs. Ferroelectric polarization domains generally reconfigure to reduce the total energy. Modification of the surface boundary conditions can enable the polarization states to undergo a reconfiguration into a conventionally unstable configuration as a response to reducing the overall depolarization field and thereby lowering the total free energy potential^13^. Depolarization field is influenced by effects pertaining to imperfect screening, reduced thickness and surface modification. Therefore, we employ a means of modifying the sample geometry of a ferroelectric BFO film through ion milling to prompt the formation of typically unstable or metastable ferroelectric domain reconfigurations.

Owing to the coupling between strain and polarization, geometric engineering presents a means to tune the physical properties and polarization of BFO nanostructures through sample geometry modification. Advances in focused ion beam (FIB) milling techniques have allowed for the fabrication of BFO nanostructures, which include nanowires, nanoislands, and nanoparticles^14–16^. Needle-shaped samples have been fabricated most commonly for Atomic Probe tomography studies of metals, polymers, semiconductors, and solid-state electrolytes^17–21^, but there are no reports of fabrication or tomographic TEM studies on perovskite needles. FIB-fabricated needle-shaped TEM specimens are commonly used for electron tomography studies to obtain three-dimensional information using a tomographic tilt series of 2D projected images. A fully tomographically reconstructed 3D structure typically requires an extended series of 2D projection images. However, the perovskite pseudocubic (PC) symmetry is advantageous for tomographic analysis as it reduces the need for an extensive tilt series. Instead, high-symmetry PC projections, (100), (110)_,_ and (010), provide the fundamental physical and polarization information, thus minimizing the need for an extensive tilt series while also avoiding the missing wedge problem^22^.

Here, the domain architecture at the apex of a BFO nanoneedle is explored in three dimensions to demonstrate a stable tail-to-tail CDW. We explore the interplay between surface curvature, size effect, and polarization domain configurations in a ferroelectric BFO nanoneedle using aberration-corrected scanning transmission electron microscopy (AC-STEM) and four-dimensional STEM (4D-STEM) to reveal stability mechanisms of CDWs in BFO nanoneedles. Additionally, phase-field simulations provide the stable ferroelectric domain distribution inside a needle-shaped sample of BFO to gain insight into the effect of a needle geometry on CDW stability. Our analysis demonstrates that modifying boundary conditions through controlling surface curvature and geometry can be a strategy for tuning the ferroelectric domain configurations.

## Results

Nanoneedle samples of BFO were fabricated using a Ga ion sourced FIB to mill a 200 nm epitaxially grown BFO film on a (110)-oriented TbScO_3_ (TSO) substrate. The nanoneedles were prepared such that the BFO (110) plane is visible in a TEM with no applied tilting of the specimen to be able to access the (100) and (110) projections with minimal tilting of the nanoneedle. Annular milling was used to gradually reduce the needle tip and achieve atomic sharpness^17,23^. The post-milled BFO nanoneedle examined is ~200 nm in height, with a 2:1 aspect ratio and ~12 nm tip radius. The consistent PC out-of-plane lattice constant measured by the Fast Fourier Transform pattern in different regions throughout the needle is ~4.02 Å, indicating preservation of the BFO rhombohedral phase after FIB milling.

**Fig. 1:**
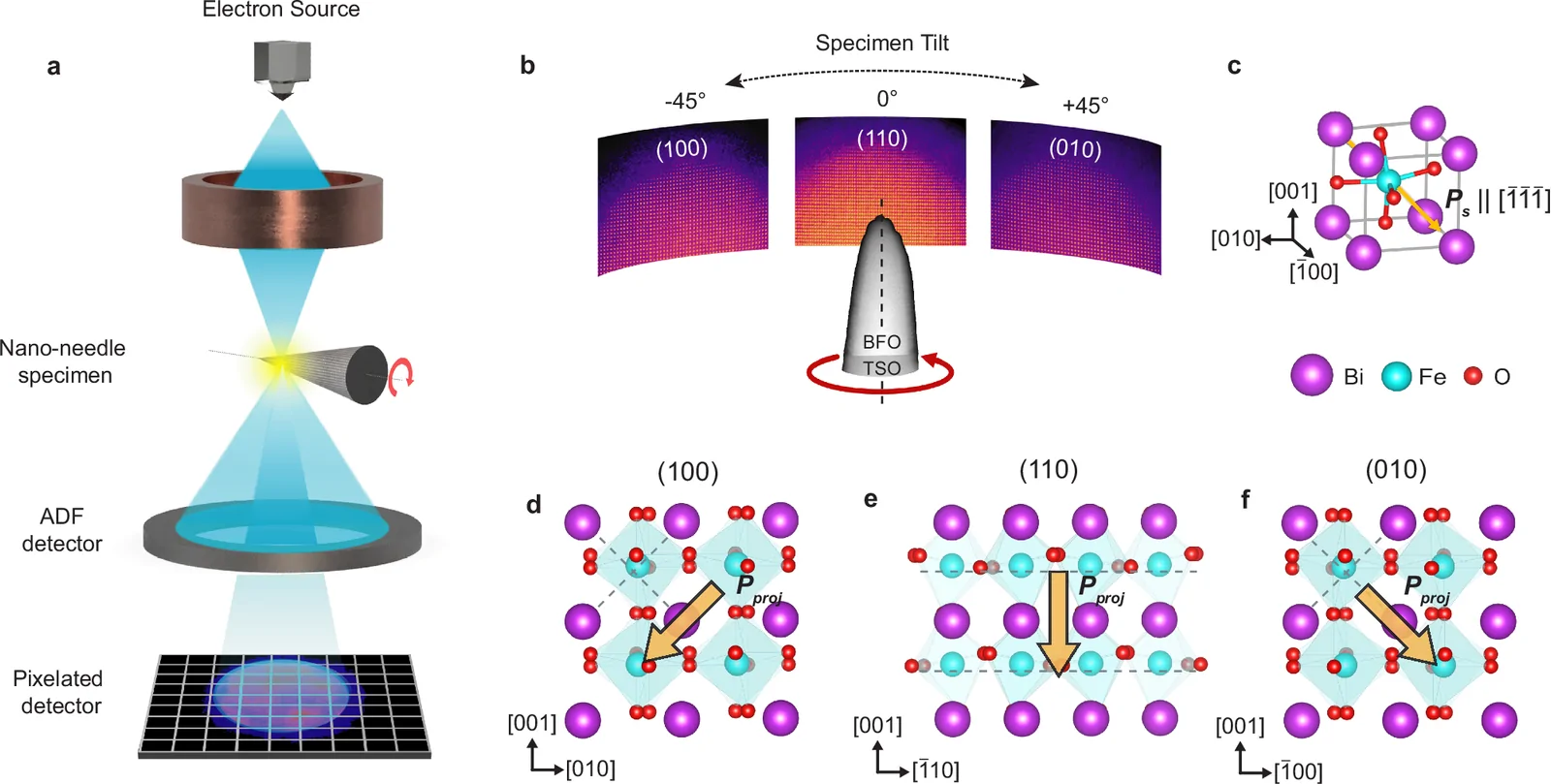
High-symmetry tilt projection series on a BFO nanoneedle. **a** Schematic of a scanning electron microscope. The nanoneedle specimen is tilted about a defined tilt axis to access low-order zone-axis projections. The ADF detector and pixelated detector collect HAADF and 4D-STEM CBED patterns, respectively. **b** Experimental STEM tilt series acquired at the nanoneedle apex, illustrating high-symmetry projections along the (100), (110), and (010) zone axes as a function of specimen tilt. **c** Three-dimensional crystal structure of BFO with the spontaneous polarization **P**_**s**_ vector oriented along the symmetry-equivalent [111] direction. **d**–**f** Atomic structural models of BFO in the pseudo-cubic lattice corresponding to the experimentally acquired projections along the (100), (110), and (010) zone axes, respectively. The lattice symmetry and projected polarization direction **P**_**proj**_, representing the image-plane component of **P**_**s**_, are highlighted.

In this study, the BFO nanoneedles were examined using an AC-STEM. Using pixelated and annular dark field detectors displayed schematically in Fig. 1a, 4D-STEM datasets and high-angle annular dark field (HAADF) STEM images were acquired. A TEM tomography sample holder with high tilting capabilities was used to collect a tilt projection series of PC high-symmetry projections (100), (110), and (010) as shown in Fig. 1b. During STEM data acquisition, the needle was tilted ±45⁰ from the (110) projection along a [001] rotation axis to reach the pair of high-symmetry (100) and (010) lattice planes. By azimuthal rotation of the needle along the crystallographic projections displayed in Fig. 1c, d, the ferroelectric polarization can be visualized around the nanoneedle. The nominal direction of the polarization in BFO is along the <111> direction, as shown in Fig. 1e. As such, the projection series allows us to infer the overall polarization at the needle tip region.

**Fig. 2:**
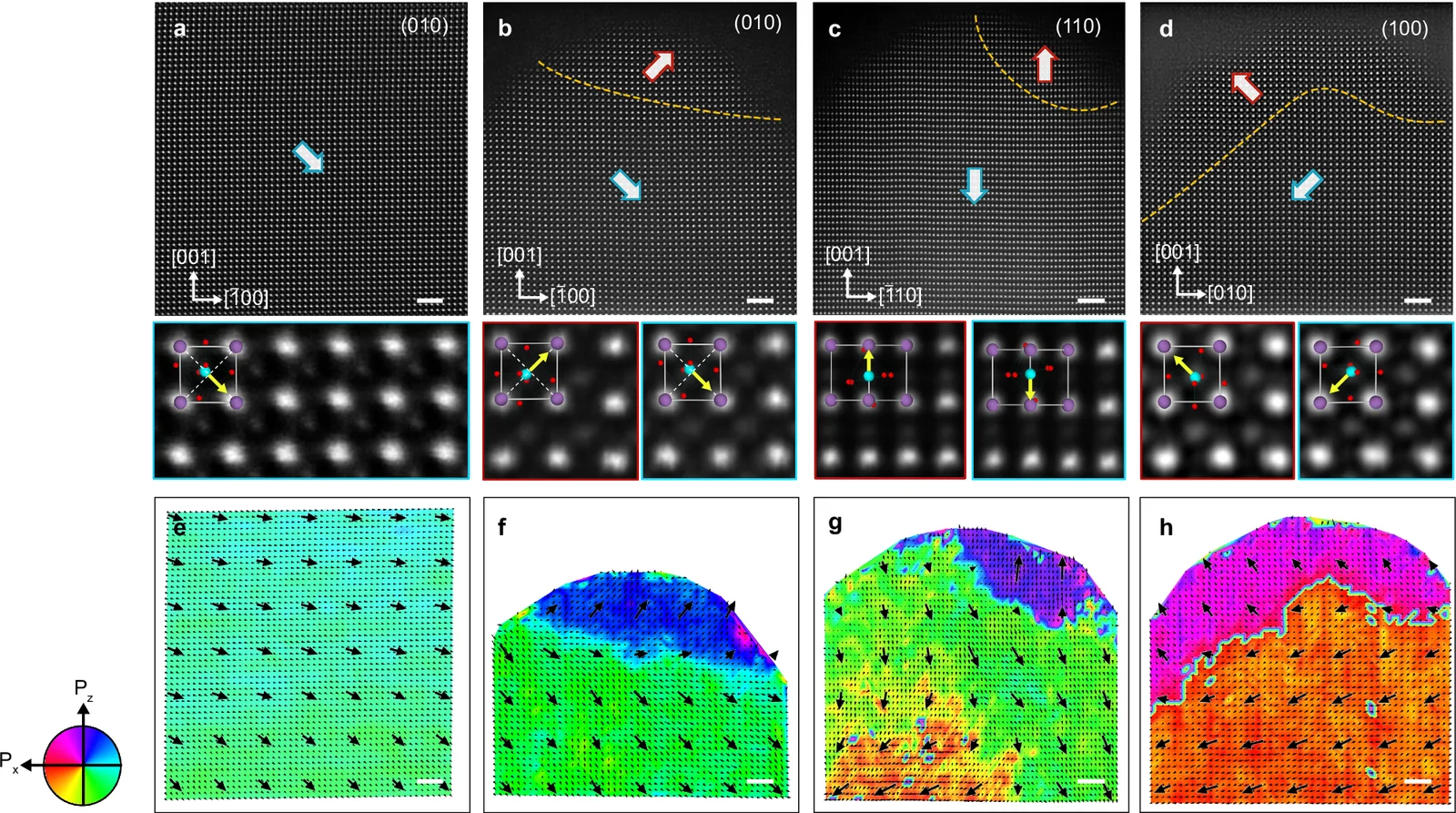
Polarization analysis of charged domains. **a** HAADF-STEM image of the pristine BFO film acquired 200 nm from the interface along the (010) projection. **b**–**d** HAADF-STEM images of the nanoneedle apex region viewed along the (010), (110), and (100) projections, respectively. Dashed orange lines mark the charged domain wall separating oppositely oriented polarization states, indicated by the red (upward) and blue (downward) arrows. Insets in panels (**a**–**d**) show the unit-cell atomic structural models overlaid on the corresponding HAADF-STEM region to highlight the projected polarization directions for each polarization state. **e**–**h** Polarization vector maps (P_x_-P_z_ components) for the pristine (**e**) and nanoneedle apex region containing a stable charged domain (**f**–**h**), obtained from the corresponding HAADF-STEM projections. Scale bars: 2 nm.

Polarization maps of the ferroelectric domain structure at the needle tip are generated from analysis of the unit cell polarization in the high-symmetry projection series. The HAADF projections used for polarization analysis, as shown in Fig. 2a–c. In the (100) and (010) projections, polarization is defined by the magnitude and direction of the displacement of the Fe cation from the centrosymmetric position formed by the four adjacent Bi atoms. For polarization determination in the (110) projection, the polarization direction is defined by the Fe displacement from the midpoint between two Bi atoms along the [001] direction. The resolved polarization vectors of the projection series, Fig. 2d–f, reveal a stable tail-to-tail CDW residing at the tip of the BFO nanoneedle. The polarization magnitude along the CDW decreases to ~30 μC/cm^2^ compared to the bulk needle polarization ~90 μC/cm^2^ in Fig. S4a. Additionally, the orientation of the polarization at the CDW is in the in-plane direction, as seen in the polarization mapping from the (100) and (010) projections. This suggests an intermediate Bloch-like polarization rotation between domains at the observed tail-to-tail CDW in the BFO nanoneedle tip region. Examination of the observed domain configuration shows that the polarization distribution and shape are asymmetric around the needle surface with an overall anisotropy. The observed asymmetry is likely influenced by the fabrication milling process that creates an imperfect needle shape with varying curvature on the surface.

**Fig. 3:**
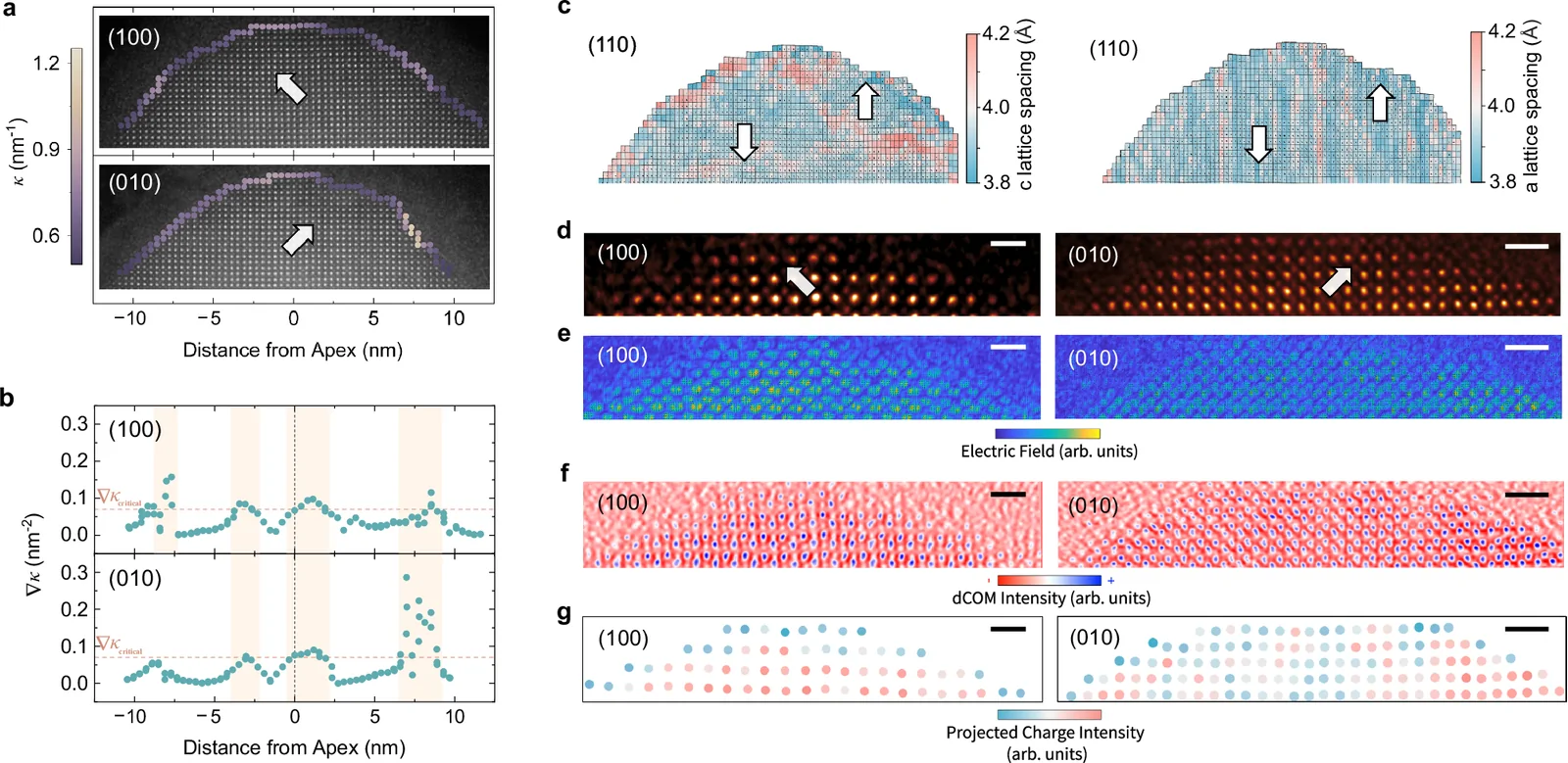
Curvature-driven surface polarization and charge redistribution at the nanoneedle apex. **a** Surface curvature extracted from B-spline fitting of the (100) and (010) surface, showing regions of enhanced surface curvature k along the apex surface. **b** Spatial variation of the curvature gradient ∇k along the surface. The dashed line denotes the threshold for polarization rotation; shaded regions mark the areas where ∇k exceeds this threshold. **c** Unit-cell resolved lattice parameters along the (110) projection, revealing coupled variations in out-of-plane lattice parameters at the charged domain wall. **d** Simultaneously captured ADF images at the nanoneedle tip along the (100) and (010) projections. **e** Reconstructed electric field maps in the same regions, showing strong field modulation near the regions of high surface curvature. **f** Derived charge density distribution, highlighting charge accumulation correlated with curvature-driven surface terminations and polarization rotation. **g** Projected charge intensity at the Fe cation sites, obtained via Voronoi integration, revealing site-specific charge asymmetry across projections. Scale bars: 1 nm.

To gather more insight into the effect of the modified surface boundary conditions on polarization stability near the nanoneedle tip, surface curvature *κ* was analyzed along the tip surface. The measured *κ*, displayed in Fig. 3a, was calculated by initially fitting a B-spline parametric curve along the 2D Gaussian fitted surface atoms at the needle surface^24^. The surface curvature was then calculated using the Frenet parametric curvature equation, where *κ* can then be expressed from the unit tangent vector rotation at a unit arc length *s*. This method for measuring curvature also allows for localized curvature variations along the surface to be observed. The B-Spline fitting results of the surface atom positions at the tip region are shown in Fig. S5. The derived surface curvature magnitude in Fig. 3a demonstrates regions of high surface curvature for both (100) and (010) projections. The distribution of high surface curvature regions is noted as symmetric and asymmetric along the (100) and (010), respectively. The asymmetry in surface curvature is clearly demonstrated using the calculated curvature gradients, *dκ/ds*, labeled as $\nabla \kappa$ in Fig. 3b. Additionally, the polarization is observed to point normal to the region of largest $\nabla \kappa$ for both projections. This suggests asymmetrical positions of high $\nabla \kappa$ regions may enable the rotation of polarization and influence the size of the polarization domains that led toward the stability of charged domains in BFO nanoneedles.

To observe how curvature variation and asymmetry that are led by high $\nabla \kappa$ function to stabilize a CDWs, the surface of the nanoneedle was polished by Ar ion milling to reduce the amorphous region and smooth the surface. The result was a nanoneedle of BFO with a symmetric and lower κ and $\nabla \kappa$, as shown in Fig. S6. Most notably, the$\,\nabla \kappa$ symmetry is uniform with no regions of high $\nabla \kappa$ comparable to the asymmetric as-milled surface. The polarization map at this lower and uniform $\nabla \kappa$ demonstrates a destabilized and therefore nonexistent CDW. Instead, the polarization reconfigures to a domain that closely resembles an unusual nanodomain configuration observed around CDWs in a 20 nm BFO/TSO thin film^25^. The unusual domain state is characterized by the preserved pseudocubic perovskite structure with an in-plane oriented polarization. The unusual nanodomain configuration emerges in response to intermediate strain conditions relative to the strain conditions necessary to stabilize CDWs. This suggests the polarization rotation is sensitive to changes in the lattice structure, where the strain conditions in the low $\nabla \kappa$ needle surface are not entirely suitable for stabilizing a CDW. Comparison of the polarization domains in the low $\nabla \kappa$ nanoneedle and the reported thin film alludes that the unusual domain exhibits a Neel-type polarization rotation. Therefore, the high $\nabla \kappa$ surfaces enable a transition from Neel to Bloch-like polarization rotation, where the Bloch-like rotations are produced by the increase in strain conditions inside the lattice.

The residing high $\nabla \kappa$ can therefore generate large strain gradients that inherently support the formation of CDWs in the BFO nanoneedle. The high $\nabla \kappa$ surfaces are expected to generate strong strain gradients that are not present in the polished surface or low $\nabla \kappa$ surfaces. The a and c lattice spacing was measured to gather insight into the enhancements in strain that may reside from a high $\nabla \kappa$. Fig. 3c shows a unit-cell map with the corresponding lattice spacings along the (110) projection. A localized expansion out-of-plane lattice spacing along the CDW is observed that cannot be attributed by free-surface relaxation alone. Instead, the c lattice expansion suggests that the CDW formation is coupled with a flexoelectric effect. Therefore, large $\nabla \kappa$ can drive flexoelectric polarization effects and promote the formation of CDWs. Based on previous Landau-Ginzburg-Devonshire (LGD) continuum phase field modeling in ferroelectric perovskites, it has been shown that the flexoelectric coupling can promote a Bloch-like polarization character^26^. Therefore, the flexoelectric effect induced by high $\nabla \kappa$ at our nanoneedle surface agrees with our observation of Bloch-like polarization rotation in the corresponding stabilized CDW.

To estimate the critical $\nabla \kappa$ that enable the polarization rotation and formation of CDWs, the symmetric Ar milled nanoneedle low $\nabla \kappa$ surface was used as a reference to measure the critical curvature gradient for the CDW formation. The maximum curvature gradient of 0.05 nm^−2^ in the Ar milled uniform surface was not enough for CDW formation, this curvature gradient value is treated as an upper bound for unusual domain formation. Moreover, on the CDW stabilized needle tip, the minimum curvature gradient measured within a polarization rotated region is 0.07 nm^−2^. Therefore, 0.07 nm^−2^ is estimated as the critical curvature gradient for enabling strong strain gradients that further active polarization rotation that lead to the formation of CDWs.

In addition to developing unique surface boundary conditions, the asymmetrical milling processing that create high $\nabla \kappa$ regions along the nanoneedle surface can additionally expose FeO_2_ terminations by removal of surface Bi atoms, that would generate surface charge imbalance., shown in Fig. S7. We approximate a critical Ga-FIB milling glancing angle of roughly 38° from the tilt axis where the FeO_2_ terminations are exposed as presented in Fig. S8. Therefore, the degree of surface curvature influences the amount of exposed surface terminations from the ion milling process. Higher surface curvatures can contribute to negative charges at the surface by exposing FeO_2_ surface terminations due to Bi vacancies forming a non-stoichiometric surface^27–29^. The exposed charged terminated surfaces modify the electrostatics at the tip where in response, the polarizations could rotate to the lowest energy surface termination to compensate for the bound polarization charges that assist to stabilize charged domains.

Charge density imaging derived from 4D-STEM datasets can reveal variations in charge density at the nanoneedle apex. The atomic charge density and electric field maps derived from the 4D-STEM dataset are shown in Fig. 3e and f. The electric-field maps represent the local electric fields derived using the center of mass shift for each scanned diffraction pattern^30–32^. The charge-density image in Fig. 3f was constructed by applying Gauss’s law to the electric-field map and contains a distribution of positive and negative charges. The contributions from the nuclear charge result in a positive charge, and the core and valence electron contributions result in a negative charge. Figure 3g displays projected Fe charge intensity derived from the integrated intensity of individual atomic Fe charge sites. To account for the reduction in thickness toward the apex, the projected charge intensity was normalized to the simultaneously captured ADF images, since ADF Z-contrast is proportional to sample thickness. The Bi site projected charge was omitted in this analysis because the ADF Z-contrast variation due to thickness effects is significantly larger than the thickness effect on the Fe Z-contrast, and therefore may unintentionally skew the projected charge. From the projected charge intensity, a relatively symmetrical charge distribution is observed in the (100) projection, whereas a strong asymmetric charge distribution is observed in the (010) projection. The observed reduction in in-plane polarization at the (100) projected surface may be associated with the reduced projected Fe charge to avoid a strongly charged surface. Moreover, the region of high projected charge in (010) projection is in the vicinity of the high $\nabla \kappa$ region. The high projected charge may be explained as the accumulation of positive bound charge from the upward polarization that is efficiently screened by the exposed FeO_2_ surface terminations in this high $\nabla \kappa$ region. As a result, surface terminations could play an additional role toward the electrostatic stability of CDWs.

**Fig. 4:**
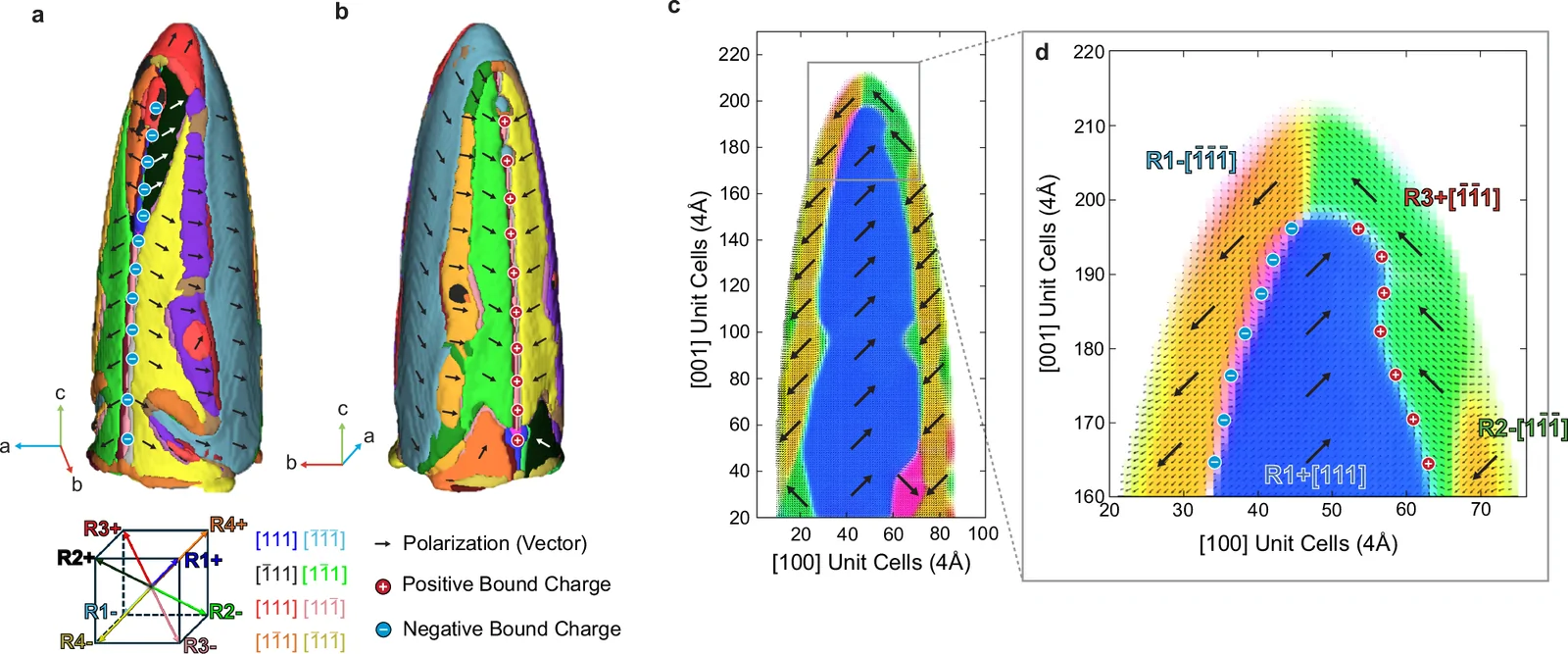
Phase-field simulations of the domain evolution in a BFO nanoneedle geometry. **a**, **b** Three-dimensional phase-field simulation of polarization domain morphology within a BFO nano-needle. Panel (**b**) shows the same structure rotated by 90°C. Colored vectors denote the symmetry-equivalent [111] polarization directions in the pseudo-cubic lattice. **c** Cross-section map of the unit-cell polarization, revealing needle-geometry induced formation of charged domain walls. **d** Magnified view of the apex region, showing domain variants and associated bound charges at the domain walls. Black arrows label the direction of polarization. Positive and negative bound charges at the charged domain walls are indicated by the red and blue markers, respectively, emerging from the polarization divergence at the domain boundaries.

Phase field simulations were utilized to investigate the formation mechanisms of CDWs within BFO nanoneedles. Starting from a single uniform domain to mimic the experimental sample before milling, we find that the resulting domain structure maintains the initial domain in the interior of the needle with polarization along the [111], with the exterior decomposing into several domains to compensate for the open-circuit electric boundary condition. The measured surface curvature is 0.83 nm^−1^, which is comparable to the milled nanoneedle surface with a CDW. The formation of CDWs is mainly a competition between electrostatic energy at the surface, driving the polarization to be parallel to the surface, electrostatic energy at the domain walls, driving the polarization to be head to tail and intrinsic chemical stability of polarization (Landau Energy), which drives the polarization along the <111> direction. From Fig. 4a and b, which demonstrate the 3-D domain structure, we find the polarizations near the surface align parallel to the surface, and arrange to compensate for the depolarization field produced by the interior [111] polarization domain. Experimentally, this phenomenon is observed in Fig. S4 by the reduced in-plane coefficient of the polarization at the needle surface. The competing driving forces allow the formation of CDWs at the surface near the (110) planes, where the electrostatic effects from the interior [111] domain are most prominent. In addition to the 3-D domain structures, we further investigate by taking 2-D slices along the (010) plane near the center of the nanoneedle (Fig. 4c, d). In the 2-D slice, we again see the interior [111] polarization domain along the polarization oriented parallel to the nanoneedle surface on the exterior. The combination of the interior domain remaining along the [111] direction and the exterior domains aligning parallel to the surface causes the formation of CDWs between the interior and exterior domains.

## Discussion

The unique nano needle geometry allows competition among Landau, electrostatic, and elastic energies, leading to the formation of a tail-to-tail CDW in nanoneedles. Experimental observations involved electrically active boundary conditions, while simulations explored a limiting case of open circuit boundary conditions to understand the nature of charged domain wall formation in BFO nanoneedles. Ultimately, the driving mechanisms for the stability of charged domains aim to decrease the depolarization field produced at the surface and CDW. From the experiment, surface terminations can mitigate surface depolarization, and phase-field simulations demonstrate that charged domains can be stabilized through domain-wall reconstruction to reduce the depolarization field within the BFO nanoneedle. The BFO polarization domains are constrained to the <111> family of directions, which limits the domains to only eight possible directions and makes domain wall reconstruction effective toward producing charged domains. Therefore, this property is essential for the formation of CDWs in BFO.

The decrease of the needle tip radius toward the surface results in a reduction in the cross-sectional area and increased strength of the depolarization field at the tip region^33–35^. The needle tip region creates electrostatic conditions that strongly influence the polarization configuration. Generally, the depolarization field is compensated by ferroelectric domain structures to decrease the overall electrostatic energy. As such, the strong depolarization field at the needle tip acts as a driving force to reconfigure the polarization distribution. Variations in surface curvature could affect the distribution of the depolarization field, thereby influencing the reconstruction of domain configurations. Similar behavior has been reported in the previous studies on thin films of BFO grown on TSO, where domain walls form to suppress the effect of the depolarization field due to the surface and BFO/TSO interfacial bound charges^34^. Similarly, the domains in a nanoneedle can perform domain reconstruction to compensate for surface charges and to limit electrostatic stray fields that may have increased due to the increase in size effects at the needle tip^7^.

The effects of needle geometries are distinguished from the geometric effects of other nanostructures by consideration of differences between surface area, curvature, and geometric aspect ratio. The high aspect ratio of nanoneedles results in a high surface area-to-volume ratio, especially at the tip, unlike planar thin-film samples. The higher surface area and curvature in nanoneedles facilitate the presence and exposure of surface terminations that aid in polar surface stability^27,36^. Moreover, greater surface curvature can further support the stability of CDWs through its influence on strain. Specifically, regions of higher surface curvature possess increased surface energy from the large surface-to-volume ratio. The reduction of surface energy via surface strain relaxation can affect the stability of polarization domains. Polarization and strain coupling can influence the total free energy in domain stabilization and polarization distribution by changing the local anisotropy of the fields^5^. The high aspect ratio of nanoneedles also allows for a sufficient distance from the substrate to reduce substrate clamping effects toward the needle tip, further promoting strain relaxation. The greater strain relaxation in nanoneedles could allow ferroelectric domain structures to rotate and reorient with more freedom and could assist in forming CDWs.

Sample aspect ratio modification in BFO through ion beam milling presents a method to stabilize conventionally unstable ferroelectric domain states. This approach offers more control of geometric effects than previous approaches to stabilizing CDWs in ferroelectric BFO nanoislands^6,7,30,37^. In BFO nanoisland studies, the nanoisland samples consisted of BFO self-assembled islands on conductive material, either a LaSrMnO_3_ buffer layer or on Nb/Fe doped SrTiO_3_ substrates, allowing for an applied voltage bias, interfacial built-in potentials, or donor/acceptor doping to help stabilize CDWs. In contrast, the studied BFO nanoneedles are fabricated on an insulating TSO substrate that does not allow the previously mentioned stability mechanisms to be accessible. Moreover, the BFO nanoneedles contained no surface-reconstructed layers (as observed in the BFO nanoislands) that could provide additional compensating surface charges. Instead, the higher aspect ratio of BFO nanoneedles allows for a more exposed surface that increases the number of exposed surface terminations to compensate for surface-bound charges.

In conclusion, the polarization configurations at the tip of an atomically sharp BFO nanoneedle were examined in three dimensions by resolving the unit cell polarization along low-order zone axes. The polarization analysis demonstrated a stable tail-to-tail CDW near the vicinity of the needle tip. The stability and formation of CDWs were explored through the lens of surface curvature and needle geometry. Phase-field simulations highlighted the interplay between electrostatic energies in the bulk and surface in stabilizing the CDWs. This study suggests that the stability and formation of CDWs are influenced by the electrostatic, elastic, and size effects governed by the surface curvature and the needle geometry. The findings in this work extend the strategies for forming charged domains in ferroelectrics by using a top-down sample preparation approach to modify the surface boundary conditions. The stability of charged domains through boundary-condition engineering enhances their potential for applications in high-density, low-power-consumption nanoelectronics.

## Methods

### Sample preparation

BFO nanoneedle samples were fabricated using a Ga source Tescan GAIA-3 GMH FIB-SEM. Trenching was performed at 20 kV operating voltage and 2.9 nA current. Annular beam milling was performed with a starting voltage of 30 kV, 600pA and then reduced to 15 kV, 141pA followed by cleaning at 3 kV, 95pA. Following fabrication, a Fischione Nanomill was used for additional cleaning and reduction of the amorphous layer at an operation voltage of 600 eV under liquid N2.

### Characterization

The STEM analysis was conducted using a JEM-ARM300F Grand ARM S/TEM at 300 kV operating voltage. HAADF images and 4D-STEM datasets were acquired using a High-Angle Triple-Axis tomography holder from Mel-Build. 4D-STEM datasets were acquired with 32 mrad convergence angle with a 15pA beam current. A Gatan K2 IS camera running at 1200 fps was used to capture the 4D STEM datasets. The electric field is calculated from the 4D STEM data based on the relative shift of the center-of-mass (COM) of each CBED pattern. The center of the CBED patterns is taken to be the average COM of the entire 4D STEM dataset. The divergence of the center-of-mass (dCOM) image is proportional to the local charge density. Images were filtered with Gatan Digital Micrograph software. Data analysis was performed using Python packages (Atomap and Temul)^38,39^ in addition to custom Matlab code.

### Curvature analysis

The surface atoms were selected, and the atomic positions were fitted with a 2D Gaussian using the Atomap Python package. To measure the curvature, the surface atoms were fitted and calculated using the open-source Fiji package as part of the ImageJ2 program.

## Supplementary information

Supplementary Information

Transparent Peer Review file

## Data Availability

The essential data in this study have been deposited in the Zenodo database under accession code https://doi.org/10.5281/zenodo.21019720 for public access. Additional data is available from the authors upon reasonable request.

## References

[1] Bednyakov, P. S. et al. Formation of charged ferroelectric domain walls with controlled periodicity. *Sci. Rep.* **5**, 15819 (2015). 10.1038/srep15819 PMC4626787 26516026

[2] Bednyakov, P. S. et al. Physics and applications of charged domain walls. *npj Comput Mater.* **4**, 65 (2018).

[3] Meier, D. et al. Anisotropic conductance at improper ferroelectric domain wall. *Nat. Mater.* **11**, 284–288 (2012). 10.1038/nmat3249 22367003

[4] Sluka, T. et al. Free-electron gas at charged domain walls in insulating BaTiO3. *Nat. Commun.* **4**, 1808 (2013). 10.1038/ncomms2839 PMC3674246 23651996

[5] Gaididei, Y., Kravchuk, V. P. & Sheka, D. D. Curvature Effects in Thin Magnetic Shells. *Phys. Rev. Lett.* **112**, 257203 (2014). 10.1103/PhysRevLett.112.257203 25014827

[6] Ma, J. et al. Controllable conductive readout in self-assembled, topologically confined ferroelectric domain walls. *Nat. Nanotech* **13**, 947–952 (2018). 10.1038/s41565-018-0204-1 30038370

[7] Chen, M. et al. Stabilization of ferroeleastic charged domain walls in self-assembled BiFeO3 nanoislands. *J. Appl. Phys.* **128**, 124103 (2020).

[8] Li, Z. et al. High-density array of ferroelectric nanodots with robust and reversibly switchable topological domain states. *Sci. Adv.* **3**, 1700919 (2017). 10.1126/sciadv.1700919 PMC5562417 28835925

[9] Lu, H. et al. Electrical tunability of domain wall conductivity in LiNbO3 thin films. *Adv. Mater.* **31**, 1902890 (2019). 10.1002/adma.201902890 31588637

[10] Ross, A. et al. Geometric Control of Domain Structure Stability in Ferroelectric Nanotubes. *Adv. Electron. Mater.* **8**, 2200132 (2022).

[11] Hertel, R. Curvature-Induced Magnetochirality. *SPIN* **03**, 1340009 (2013).

[12] Sheka, D. D. et al. Nonlocal chiral symmetry breaking in curvilinear magnetic shells. *Commun. Phys.* **3**, 128 (2020).

[13] Siannas, N. et al. Metastable ferroelectricity driven by depolarization fields in ultrathin Hf_0.5_Zr_0.5_O_2_. *Commun. Phys.* **5**, 178 (2022).

[14] Morelli, A. et al. Ferroelectric nanostructures fabricated by focused-ion-beam milling in epitaxial BiFeO_3_ thin films. *Nanotechnology* **22**, 26503 (2011). 10.1088/0957-4484/22/26/265303 21576790

[15] Zhang X. et al. Synthesis and characterization of highly ordered BiFeO3 multiferroic nanowire arrays. *Progress in Solid State Chemistry* **33**, 10.1016/j.progsolidstchem.2005.11.027 (2005).

[16] Hasan, M. et al. A soft chemical route to the synthesis of BiFeO3 nanoparticles with enhanced magnetization. *Materials Research Bulletin* **73**, 10.1016/j.materresbull.2015.09.007 (2016).

[17] Hernandez-Saz, J. et al. A methodology for the fabrication by FIB of needle-shape specimens around sub-surface features at the nanometre scale. *Micron* **43**, 643–650 (2012).

[18] Herrera, M. et al. Low dose electron tomography of novel nanocomposites for additive manufacturing. *Polym. Test.* **128**, 108232 (2023).

[19] Stegmaier, S. et al. Nano-Scale Complexions Facilitate Li Dendrite-Free Operation in LATP Solid-State Electrolyte. *Adv. Energy Mater.* **11**, 2100707 (2021).

[20] Chang, Y. et al. Ti and its alloys as examples of cryogenic focused ion beam milling of environmentally-sensitive materials. *Nat. Commun.* **10**, 942 (2019). 10.1038/s41467-019-08752-7 PMC6391424 30808943

[21] Xu, R. et al. Three-dimensional coordinates of individual atoms in materials revealed by electron tomography. *Nat. Mater.* **14**, 1099–1103 (2015). 10.1038/nmat4426 26390325

[22] Liu, Y. et al. Isotropic reconstruction for electron tomography with deep learning. *Nat. Commun.* **13**, 6482 (2022). 10.1038/s41467-022-33957-8 PMC9617606 36309499

[23] Kant, K. & Losic, D. *FIB Nanostructures* **20**, Springer (2013).

[24] Pressley A., How much does a curve curve? In: *Elementary Differential Geometry* (Springer Undergraduate Mathematics Series). Springer, London (2010).

[25] Linze, L. et al. Atomic Scale Structure Changes Induced By Charged Domain Walls in Ferroelectric Materials. *Nano Lett.* **13**, 5218–5223 (2013). 10.1021/nl402651r 24070735

[26] Eliseev, E. et al. Structural phase transitions and electronic phenomena at 180-degree domain walls in rhombohedral BaTiO_3_. *Phys. Rev. B.* **87**, 054111 (2013).

[27] Efe, I. et al. On the happiness of ferroelectric surfaces and its role in water dissociation: The example of bismuth ferrite. *J. Chem. Phys.* **154**, 024702 (2021). 10.1063/5.0033897 33445895

[28] Garrity, K. et al. Ferroelectric surface chemistry: First principles study of PbTiO_3_ surface. *Phys. Rev. B* **88**, 045401 (2013).

[29] Gao, P. et al. Atomic mechanism of polarization-controlled surface reconstruction in ferroelectric thin films. *Nat. Commun.* **7**, 11318 (2016). 10.1038/ncomms11318 PMC4838897 27090766

[30] Han, M. et al. Shape and Surface Charge Modulation of Topological Domains in Oxide Multiferroics. *J. Phys. Chem. C.* **4**, 2557–2564 (2019).

[31] Muller-Caspary, K. et al. Atomic-scale quantification of charge densities in two-dimensional materials. *Phys. Rev. B* **98**, 121408 (2018).

[32] Gao, W. et al. Real-space charge-density imaging with sub-angstrom resolution by four-dimensional electron microscopy. *Nature* **575**, 480–484 (2019). 10.1038/s41586-019-1649-6 31610544

[33] Gao, P. et al. Possible absence of critical thickness and size effect in ultrathin perovskite ferroelectric films. *Nat. Commun.* **8**, 15549 (2017). 10.1038/ncomms15549 PMC5467161 28585548

[34] Probing Ferroic States in Oxide Thin Films Using Optical Second Harmonic Generation. *Appl. Sci*. **8**, 10.3390/app8040570 (2018).

[35] Huyan, H. et al. Direct observation of polarization-induced two-dimensional electron/hole gases at ferroelectric-insulator interface. *njp Quantum Mater.* **6**, 88 (2021).

[36] Gattinoni, C. et al. Interface and surface stabilization of the polarization in ferroelectric thin films. *Appl. Phys. Sci.* **117**, 28589–28595 (2020). 10.1073/pnas.2007736117 PMC7682414 33122429

[37] Peng, R. Understanding and predicting geometrical constraint ferroelectric charged domain walls in a BiFeO_3_ island via phase-field simulations. *Appl. Phys. Lett.* **113**, 222902 (2018).

[38] Nord, M. et al. Atomap: a new software tool for the automated analysis of atomic resolution images using two-dimensional Gaussian fitting. *Adv. Struct. Chem. Imag.* **3**, 9 (2017). 10.1186/s40679-017-0042-5 PMC5306439 28251043

[39] O’Connell E., Hennessy M. & Moynihan E. PinkShanack/TEMUL(Version 0.1.7) (2022).

