# Supplementary material for: Stabilization of charged domains in ferroelectric BiFeO3 nanoneedles
Source: Nat Commun. 2026 Aug 26;17:10190. doi: 10.1038/s41467-026-76259-z (PMC13612826; doi:10.1038/s41467-026-76259-z)
Supplement: Supplementary file 1 [file 41467_2026_76259_MOESM1_ESM.pdf]

Supplementary Information for:

Stabilization of charged domains in ferroelectric $BiFeO_3$ nanoneedles.

Francisco Guzman[1], Christopher Addiego[2], Aiden Ross[3], Moaz Waqar[1], Long-Qing Chen[3], and Xiaoqing Pan[1,2,4*]

[1.] Department of Materials Science and Engineering, University of California, Irvine, CA 92697, USA.

[2.] Department of Physics and Astronomy, University of California, Irvine, CA 92697, USA.

[3.] Irvine Materials Research Institute, University of California, Irvine, CA 92697, USA.

[4.] Materials Science and Engineering Department, Pennsylvania State University, University Park, PA 16802, USA.

* Corresponding author: xiaoqinp@uci.edu

Phase-Field Simulations:

The phase-field model for ferroelectrics was used to simulate the polarization distribution in the $BiFeO_3$ nanoneedle.[1] The temporal evolution of the polarization was described by the time-dependent Ginzburg-Landau equation

$$\frac{\partial P_i(r_i,t)}{\partial t} = -L_{ij}\frac{\delta F(t)}{\delta P_i(r_i,t)}. \quad (1)$$

Where P_i represents the total ferroelectric polarization, r_i represents the position in space, t is time and L is the kinetic coefficient related to domain wall motion. The total free energy functional F is comprised of the following energy-density terms integrated over the entire system

$$F = \int_V (f_{Landau} + f_{Gradient} + f_{Elastic} + f_{Electric})dV \quad (2)$$

A system size of $96\Delta x_1 \times 96\Delta x_2 \times 220\Delta x_3$ was used with $\Delta x_1 = \Delta x_2 = \Delta x_3 = 1nm$. Periodic boundary conditions were employed at the edges of the system. We define the nanoneedle as a half-ellipsoid with a height of 200nm and a radius of 30nm at the base. We define a 3nm amorphous layer on the surface of the nanoneedle which was formed during the FIB milling of the nanoneedle. On the bottom surface of the nanoneedle, we define a 20nm thick substrate to simulate the effect of clamping.

Following the Landau-Devonshire theory of ferroelectrics, we describe the relative stability of the ferroelectric phase in reference to the paraelectric phase in $BiFeO_3$ by a 6th-order polynomial expansion about the polarization order parameter.

$$f_{landau} = a_1(T)(P_1^2 + P_2^2 + P_3^2) + a_{11}(P_1^4 + P_2^4 + P_3^4) + a_{12}(P_1^2P_2^2 + P_1^2P_3^2 + P_2^2P_3^2)$$
$$+a_{111}(P_1^6 + P_2^6 + P_3^6) + a_{112}\left(P_1^4(P_2^2 + P_3^2) + P_2^4(P_1^2 + P_3^2) + P_3^4(P_2^2 + P_1^2)\right) + a_{123}P_1^2P_2^2P_3^2 \quad (3)$$

Where a_i, a_{ij} and a_{ijk} are coefficients related to the dielectric stiffness of the material measured under stress-free conditions. For $BiFeO_3$ these coefficients were obtained from[2] and are the following $a_1 = 4.64385 \times 10^5(T - 1103)\frac{m^2N}{C^2}$, $a_{11} = 2.29047 \times 10^8\ \frac{m^6N}{C^4}$, $a_{12} = 3.06361 \times$

$10^8 \frac{m^6 N}{C^4}$, $a_{111} = 5.99186 \times 10^7 \frac{m^{10} N}{C^6}$, $a_{112} = -3.33980 \times 10^5 \frac{m^{10} N}{C^6}$, $a_{123} = -1.77754 \times 10^8 \frac{m^{10} N}{C^6}$.

The local gradient energy density is defined by

$$f_{Gradient} = \frac{1}{2} G_{ijkl} \frac{\partial P_i}{\partial x_j} \frac{\partial P_k}{\partial x_l} \quad (4)$$

We assume an isotropic gradient energy tensor G_{ijkl} with normalized values of $G_{11} = 0.60$, $G_{12} = -0.60$, and $G_{44} = 0.60$ in Voigt notation.

The elastic energy density is defined by

$$f_{Elastic} = c_{ijkl}(\varepsilon_{ij} - \varepsilon_{ij}^{o})(\varepsilon_{kl} - \varepsilon_{kl}^{o}) \quad (5)$$

$$\varepsilon_{ij}^{o} = Q_{ijkl} P_k P_l$$

Where c_{ijkl} is the elastic stiffness tensor, Q_{ijkl} is the electrostrictive tensor, ε_{ij} is the total strain and ε_{ij}^{o} is the strain associated with the ferroelectric polarization typically called the electrostrictive strain or eigenstrain. At each timestep we solve the mechanical equilibrium equation $\nabla \cdot \sigma_{ij} = 0$ with the stress equal to $\sigma_{ij} = c_{ijkl}(\varepsilon_{kl} - \varepsilon_{kl}^{o})$. The elastic energy coefficients are for the $BiFeO_3$ $Q_{11} = 0.032 \frac{m^4}{C^2}$, $Q_{12} = -0.016 \frac{m^4}{C^2}$, $Q_{44} = 0.04 \frac{m^4}{C^2}$, $C_{11} = 3.02 \times 10^{11} \frac{N}{m^2}$, $C_{12} = 1.62 \times 10^{11} \frac{N}{m^2}$, $C_{44} = 0.68 \times 10^{11} \frac{N}{m^2}$, for the $TbScO_3$ substrate $C_{11} = 3.02 \times 10^{11} \frac{N}{m^2}$, $C_{12} = 1.62 \times 10^{11} \frac{N}{m^2}$, $C_{44} = 0.68 \times 10^{11} \frac{N}{m^2}$, for the amorphous layer $C_{11} = 2.28 \times 10^{10} \frac{N}{m^2}$, $C_{12} = 1.28 \times 10^{10} \frac{N}{m^2}$, $C_{44} = 0.65 \times 10^{10} \frac{N}{m^2}$, and for the air was set to $C_{ij} \cong 1$ which automatically satisfies the stress-free boundary conditions at the nanoneedle surface, however for numerical stability we define $C_{11} = 1 \frac{N}{m^2}$, $C_{12} = 1 \frac{N}{m^2}$, $C_{44} = 1 \frac{N}{m^2}$.

The electrostatic energy density is expressed as

$$f_{Electric} = -\frac{1}{2} \epsilon_0 \kappa_{ij}^{b} E_i E_j - E_i P_j \quad (6)$$

Where ϵ_0 is the vacuum permittivity and κ_{ij}^b is the background dielectric constant which considers the contributions to the total dielectric constant that are not related to the ferroelectric soft-mode.[3] In the $BiFeO_3$ and the amorphous layer $\kappa_{ij}^b = 10$ and in the air the $\kappa_{ij}^b = 1$.

Starting from a random polarization distribution, the equilibrium domain structure was obtained by numerically solving the time-dependent Ginzburg-Landau equation until an equilibrium state was reached. Here we define the equilibrium as a relative change of $F_{tot} < 0.02\%$ for 1000 time-steps. To account for the large elastic and dielectric inhomogeneity a Fourier spectral iterative perturbative method was employed to solve the elastic and electric equilibrium equations.[4] The phase-field calculations were preformed using the μ-Pro software.[5]

Supplemental Figures:

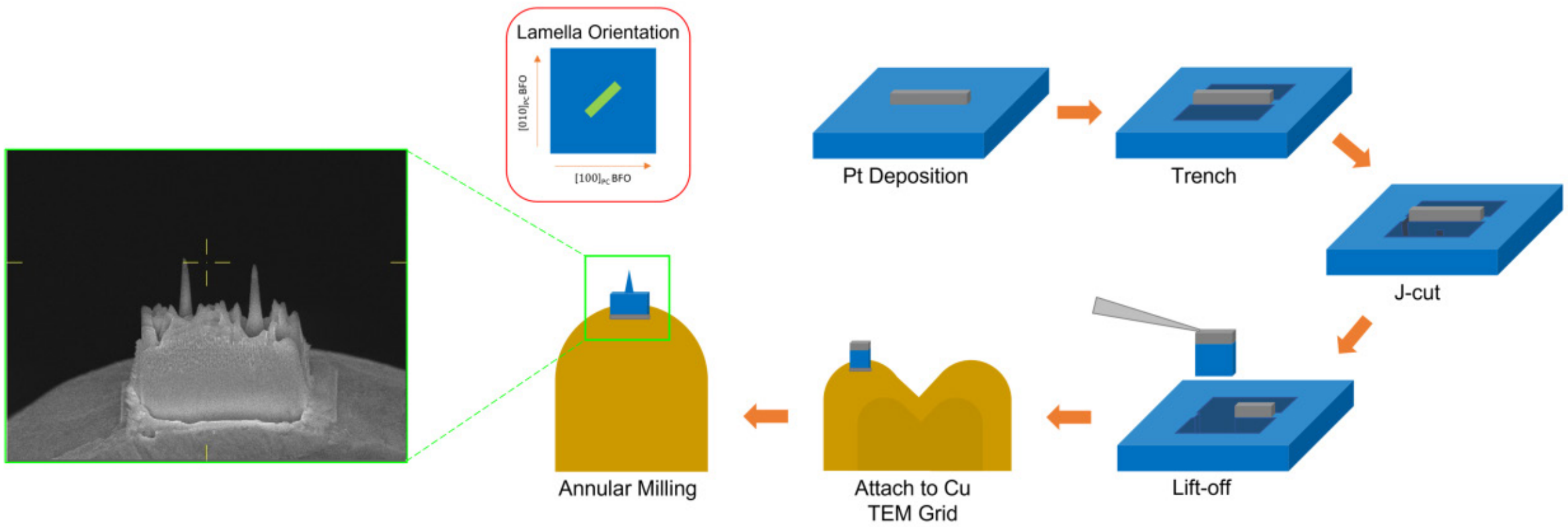

Figure S1 | Needle Fabrication. Schematic of needle fabrication using focused ion beam milling including an SEM image of lamella TEM specimen with two BFO nanoneedles.

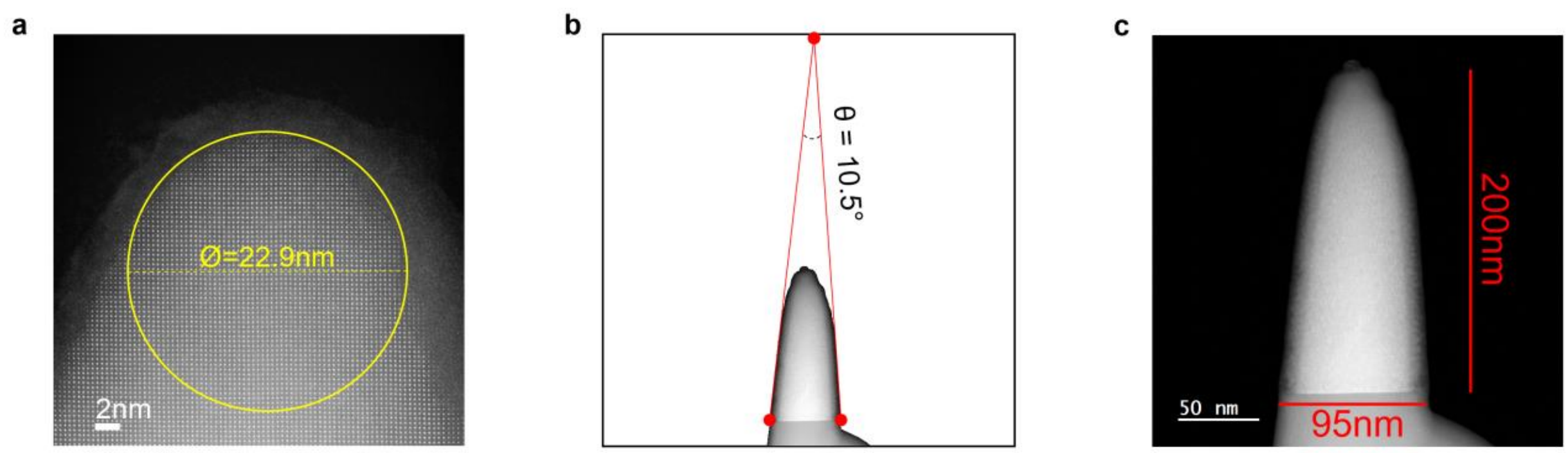

Figure S2 | Needle Parameters. (a) Tip Radius measurement (b) Needle angle θ (c) Needle height and base. Aspect ratio of approximately 2:1.

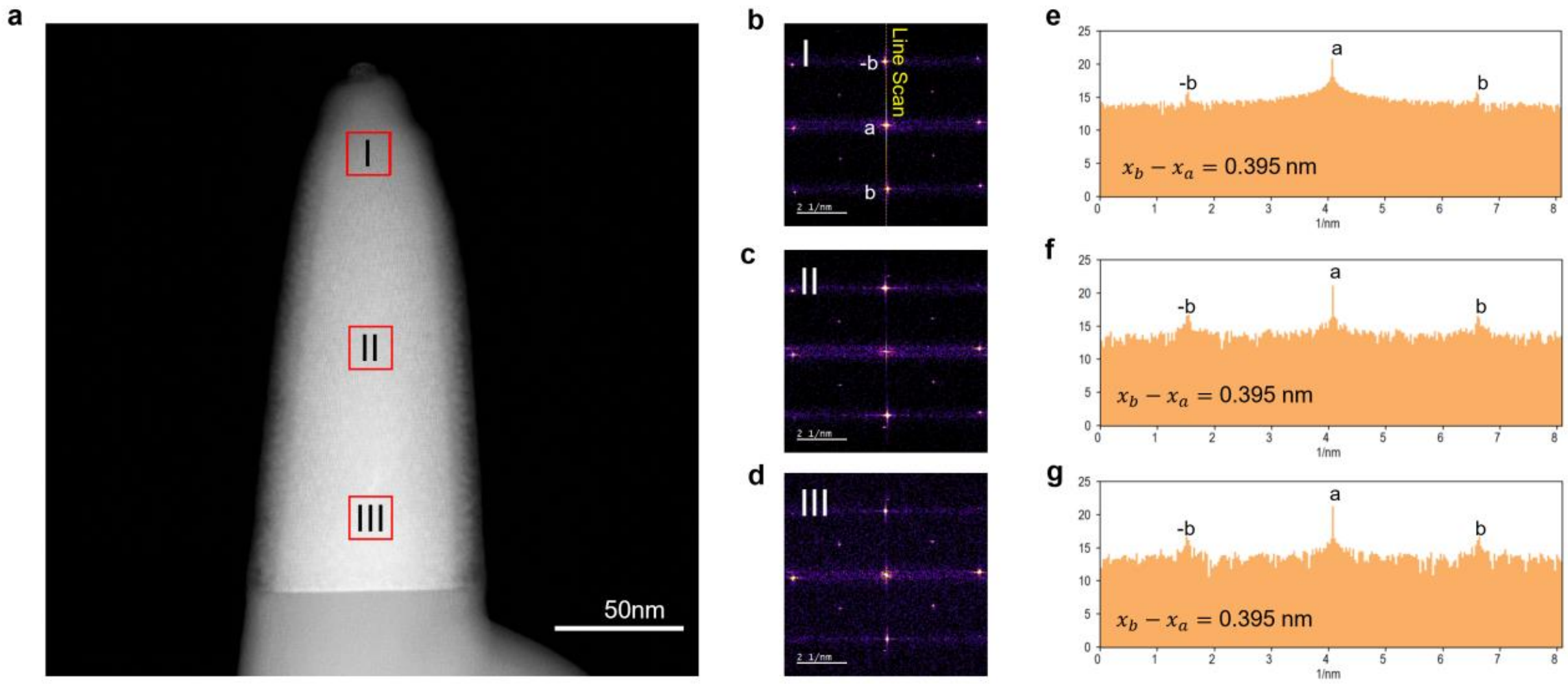

Figure S3 | BFO FFT Patterns. (a) Post annular milled BFO nanoneedle specimen. Red boxes represent regions of acquired FFT. (b,c,d) FFTs acquired at regions I,II and III in figure a. (e,f,g) FFT line scan across FFT peaks along dashed line in figure b for regions I,II and III respectively.

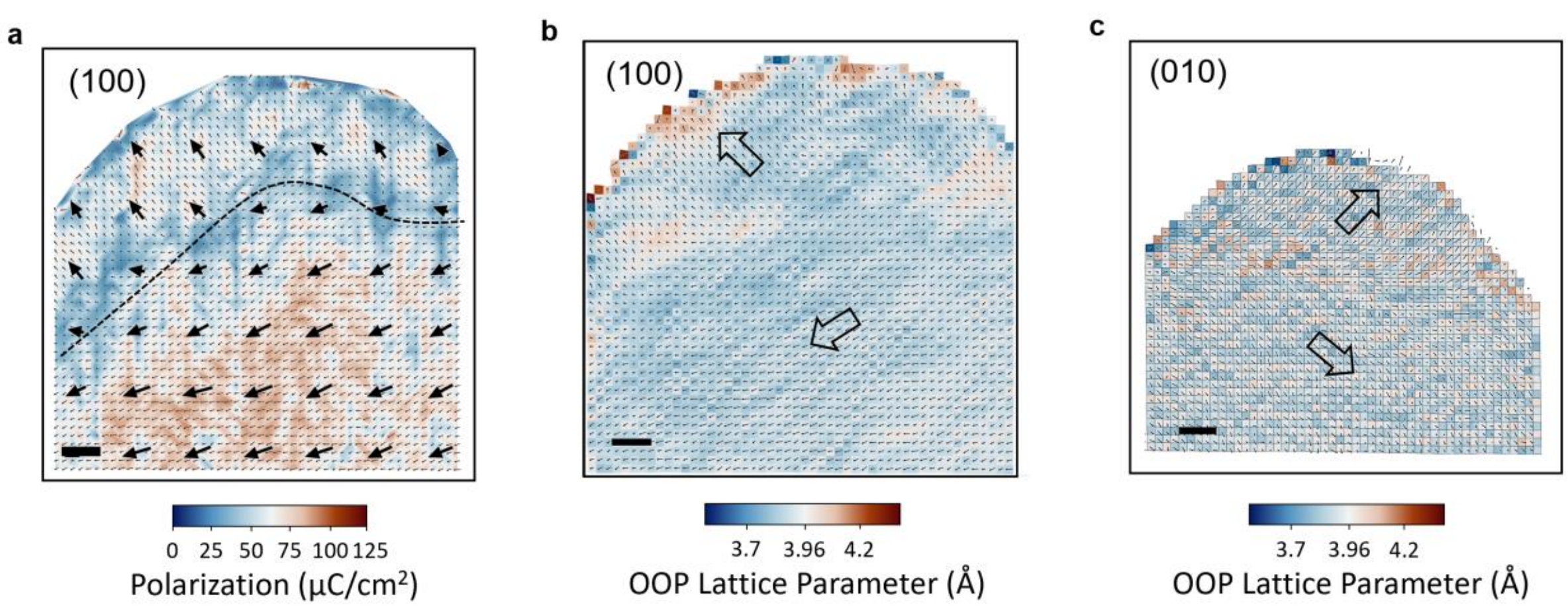

Figure S4 | Supplementary Polarization and Structure Analysis. (a) Ferroelectric polarization magnitude map of the (100) projected needle tip region. The polarization was derived based on the Born effective charges of BFO and the calculation adopted from ref. 6. Overlayed vectors represent the average magnitude and direction. (b) Unit-cell out-of-plane lattice strain measurement of (100) projection. (c) Out-of-plane lattice constant measurement of (010) projection HAADF image. Scalebar is 2nm.

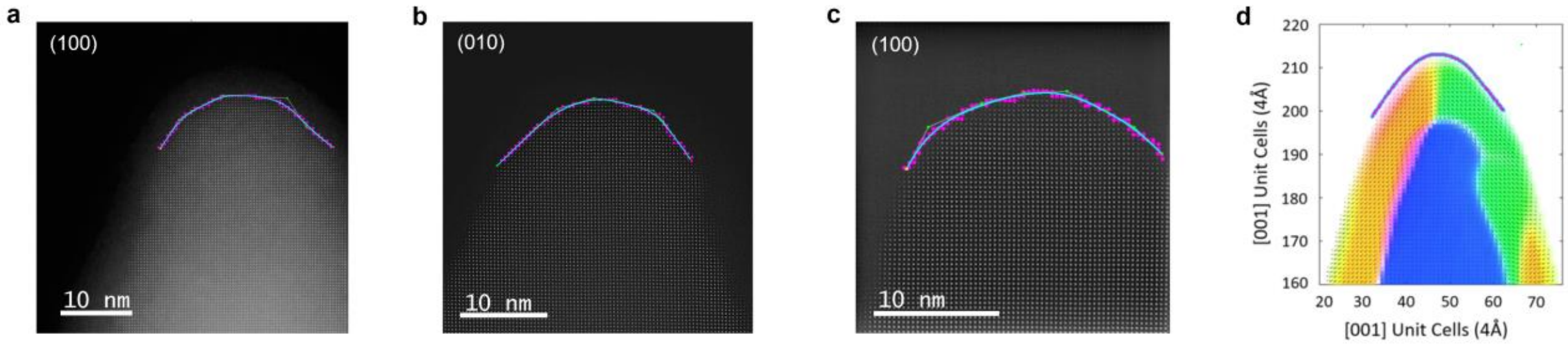

Figure S5 | Surface Curvature Determination of BFO Nanoneedle Apexes. (a,b) Parametric curve fitting of the Bi surface atom positions from the HAADF-STEM images of the BFO nanoneedle apex along the (100) and (010) projections. (c) Parametric curve fitting of the Bi surface atom positions for the Ar ion polished apex viewed along the (100) projection. (d) Parametric surface fitting of the phase-field simulated nanoneedle apex surface.

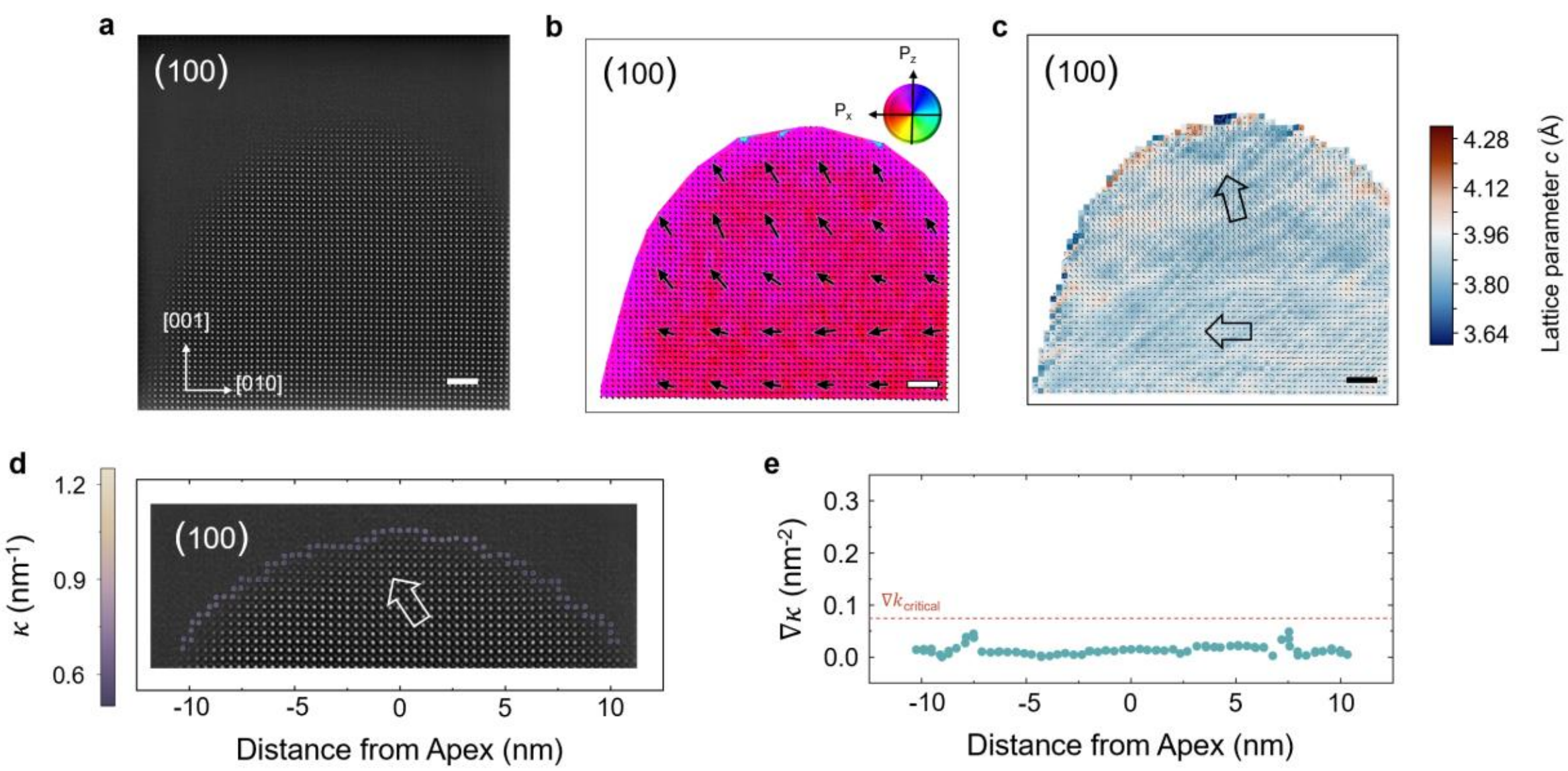

Figure S6 | Supplemental Analysis of Uniform Curvature Surface. (a) (100) projected HAADF-STEM image of a smooth nanoneedle surface. (b) Corresponding polarization vector map of figure a. (c) Out-of-plane lattice constant measurement of figure b. (d) Surface curvature of a BFO nano-needle at the apex of the nanoneedle surface of figure a, demonstrating a symmetric and overall uniform surface curvature. (c) Curvature gradient plot derived from the surface curvature, demonstrating a relatively uniform curvature graident at the nanoneedle surface. Dashed line represents the approximated critical curvature gradient for charged domain formation. Scalebar is 2nm.

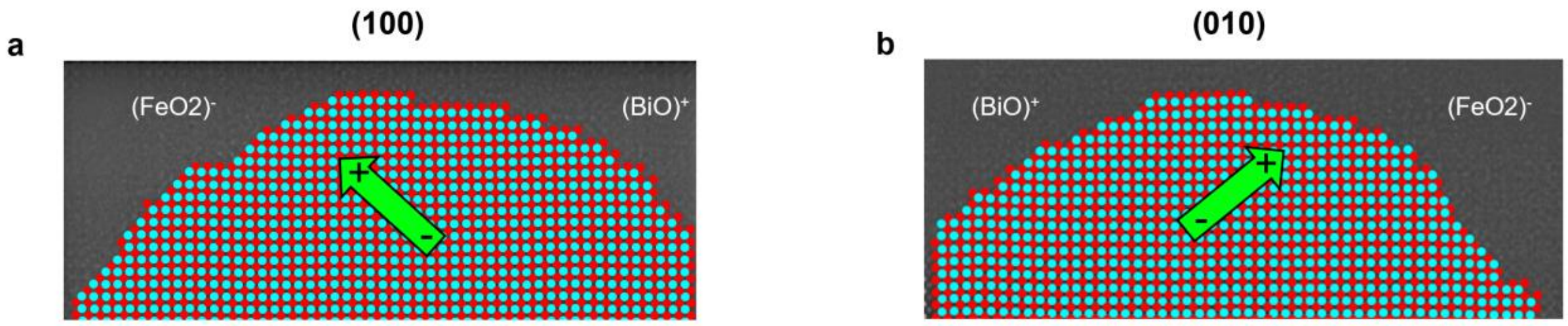

Figure S7 | Surface Terminations. (a,b) 2D-Gaussian fitted Bi and Fe atomic positions for the (100) and (010) HAADF projections at the BFO needle tip region. Higher surface curvatures expose Fe atoms on the surface leading to different terminations across the needle surface. Bi and Fe atoms are represented by the red and cyan circles, respectively.

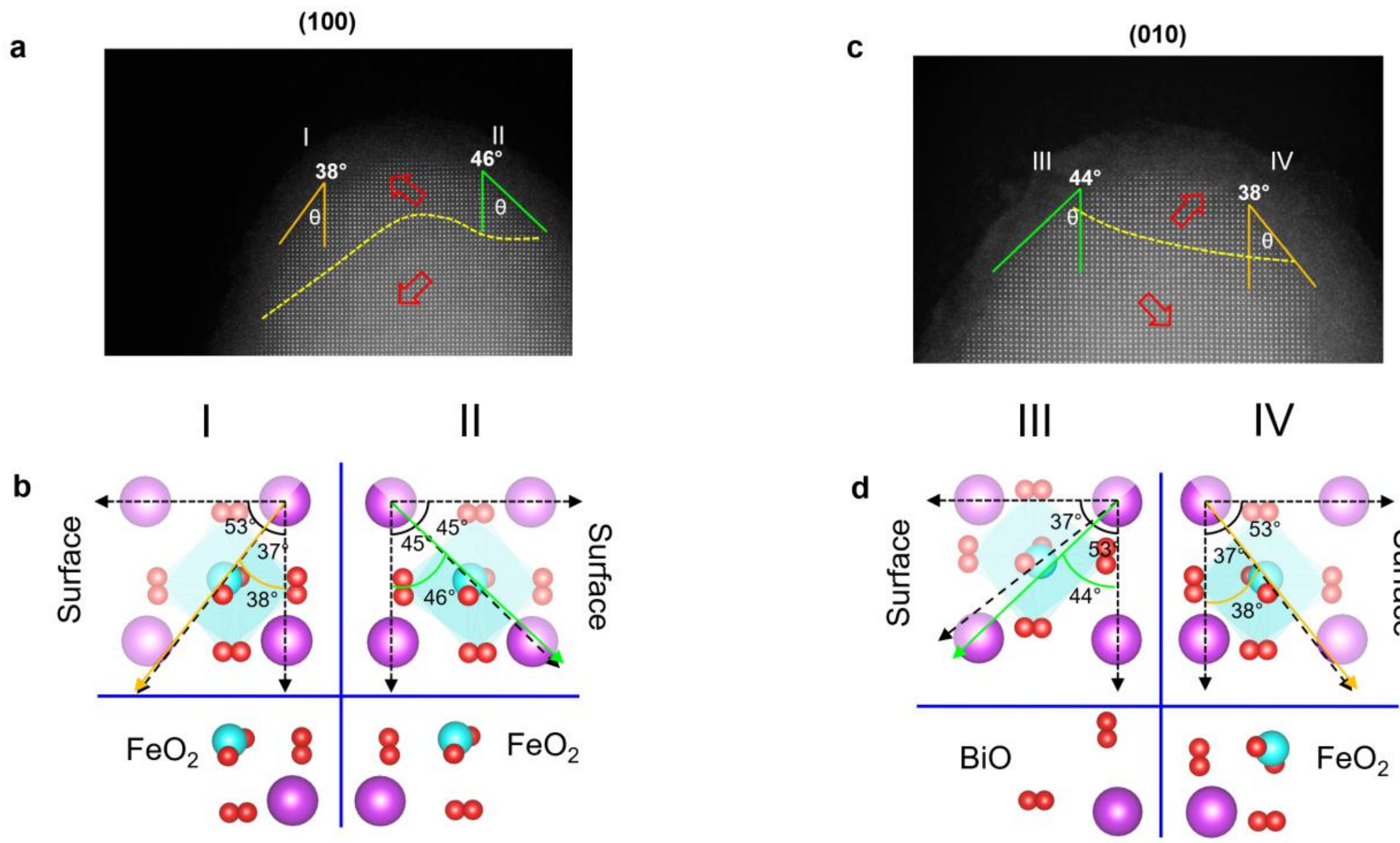

Figure S8 | Surface Angle and Terminations. (a,c) HAADF atomic images of at the tip of the BFO nanoneedle. The angle of curvature (θ) is measured in regions of the curved surface labeled I,II, III and IV. (b,d) Atomic models of a PC unit cell of BFO overlayed with measured θ value. The predicted corresponding surface termination is shown below their atomic model.

It was worth noting that some surface angles (I,II) are close to the expected angle for removal of the Fe atom, however the larger bond strength of FeO_2 compared to BiO was considered to predict the surface termination. Likely angles considerably larger than the Fe atom line path will remove the Fe atom as seen in scenario III.

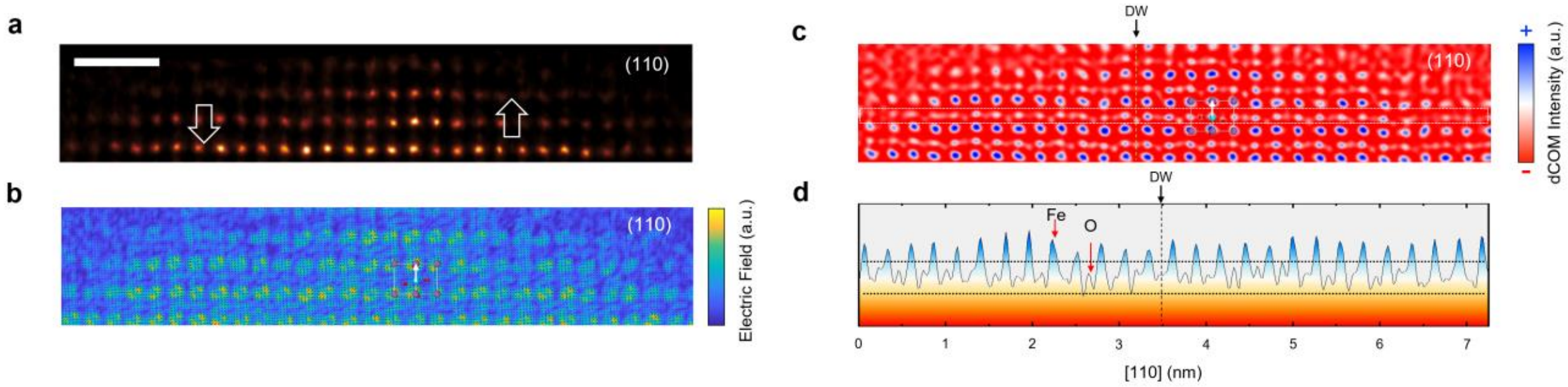

Figure S9 | Supplementary 4D-STEM analysis. 4D-STEM surface analysis for the (110) projection (a) Simultaneously acquired ADF image at the BFO nanoneedle apex region. (b) Electric field map reconstructed from a 4D-STEM dataset acquired in the region of (a). (c) Corresponding differential center-of-mass (dCOM) map derived from the electric field map, demonstrating charge at the nanoneedle surface. (d) dCOM intensity profile extracted along the dashed box in (c), showing the variation of charge across alternating Fe and O atomic columns. No apparent reduction in oxygen column intensity is observed in the anticipated location of the domain wall, suggesting no significant concentration of oxygen vacancies residing at the domain wall. Scale bar is 2 nm.

References:

1. Li, Y. L., Hu, S. Y., Liu, Z. K. & Chen, L. Q. Effect of substrate constraint on the stability and evolution of ferroelectric domain structures in thin films. *Acta Materialia* **50**, 395–411 (2002).

2. Vasudevan, R. K. *et al.* Domain Wall Geometry Controls Conduction in Ferroelectrics. *Nano Lett.* **12**, 5524–5531 (2012).

3. Levanyuk, A. P., Strukov, B. A. & Cano, A. Background dielectric permittivity: Material constant or fitting parameter? *Ferroelectrics* **503**, 94–103 (2016).

4. Wang, J. J., Ma, X. Q., Li, Q., Britson, J. & Chen, L.-Q. Phase transitions and domain structures of ferroelectric nanoparticles: Phase field model incorporating strong elastic and dielectric inhomogeneity. *Acta Materialia* **61**, 7591–7603 (2013).

5. https://mupro.co/.

6. Nelson C. *et al.*, Spontaneous Vortex Nanodomain Arrays at Ferroelectric Heterointerfaces, *Nano Letters.* **11**, 828-834 (2011)
