# Supplementary material for: Stabilization of charged domains in ferroelectric BiFeO3 nanoneedles
Source: Nat Commun. 2026 Aug 26;17:10190. doi: 10.1038/s41467-026-76259-z (PMC13612826; doi:10.1038/s41467-026-76259-z)
Supplement: Supplementary file 2 [file 41467_2026_76259_MOESM2_ESM.pdf]

nature portfolio

Peer Review File

Stabilization of Charged Domains in Ferroelectric $BiFeO_3$ Nanoneedles

Corresponding Author: Professor Xiaoqing Pan

This file contains all reviewer reports in order by version, followed by all author rebuttals in order by version.

Version 0:

Reviewer comments:

Reviewer #1

(Remarks to the Author)
The authors observed charged domain wall and investigated the surface boundary in the ending of the BiFeO3(BFO) nanoneedles by scanning transmission electron microscopy. The author also used phase-field simulations to investigate the formation mechanism of CDWs within BFO nanoneedles. While the manuscript explores an interesting topic in ferroelectric CDWs, the current version does not sufficiently demonstrate its scientific significance or provide robust data to support the conclusions. I will not recommend this paper for publication on Nature Communication unless the following major issues are addressed. Below are my specific comments.

Comment 1. The authors claimed that surface curvature plays a role in polarization domain control. The direction of the average measured polarization is within the quadrant with the highest curvature. This conclusion is insufficient unless multiple nanoneedles which have highest curvature point to different quadrants are measured. What's the initial polarization of the BFO film? Does the average polarization relevant to the initial polarization of the BFO film?

Comment 2. The authors clarified the CDW is stabilized. How to get a stabilized CDW? What's the critical condition? Under what kind of curvature? Or at what level of asymmetry? The paper currently lacks sufficient data on the manipulation of the CDW by surface curvature.

Comment 3. Can the stabilized CDW be read out or manipulated by electric field? What's the application prospects of this stabilized CDW in the specific nanoneedle structure?

Comment 4. The titles of the paper should be unified. Currently, the titles of the main text and the SI are not consistent.

Reviewer #2

(Remarks to the Author)
Overall I find this work to be of significant merit and contains the noteworthy observation of charged tail-to-tail domain walls at the tip of a BiFeO3 nanoneedle. The authors performed detailed experimental work using STEM imaging and 4D-STEM at the atomic level to provide evidence for the reasons for the domain structure formation. Polarization mapping from the STEM imaging and charge density observations from 4D-STEM are excellent tools to gain a clear understanding of the ferroelectric domain structure. Phase-field modelling supported their conclusions. There has been previous work using FIB milling showing how nanoscale geometric confinement affects ferroelectric domain structures, (e.g. Schilling, A., et al. "Morphological control of polar orientation in single-crystal ferroelectric nanowires." Nano letters 7.12 (2007): 3787-3791.), and charged domain walls in similar doped materials have been reported (e.g. Moore, Kalani, et al. "Charged domain wall and polar vortex topologies in a room-temperature magnetoelectric multiferroic thin film." ACS Applied Materials & Interfaces 14.4 (2022): 5525-5536.). However those did not use pure BiFeO3, and did not use the nanoneedle geometry. As mentioned by the authors, the sample-substrate geometry is critical in controlling the strain relaxation to govern specif domain pattern formation. The insight in this work that surface angle, and the specific associated surface charge effects can induce charged domain wall formation is notable. The experimental evidence is thorough, and though the number of observation/samples analyzed is small, the conclusions are logical and well reasoned. There is certainly enough detail provided for the community to reproduce and build on the work.

Below are specific comments that I believe will improve the interpretation and clarity of the work for the reader.

Line 48 : This should cite ‘H. Lu et al. Electrical Tunability of Domain Wall Conductivity in LiNbO3 Thin Films. Adv. Mater. 2019, 31, 1902890. https://doi.org/10.1002/adma.201902890’

Lines 54-56: This comment about domain wall surface curvature extrapolated from a different material system isn’t well justified or very helpful. Domain wall surface curvature is generally energetically unfavorable. Indeed this is why most bulk domain walls are very straight. Exploring the situations where domain walls are curved is very interesting, but that is a consequence of the sample surface geometry that forces the domain walls to adapt. This is explained well in the discussion, and would be better context to provide in the introduction.

Lines 61-62: This makes it sound like the domain structures are needles. Better to say Needle shaped samples. The most common use of needle-samples fabricated by FIB is for Atom Probe tomography. This should probably be mentioned.

Fig1 c, d: Should attempt to convey that the allowed bulk polarization direction in BFO are from the Fe, angled towards a corner Bi atom along <111>. Not parallel/perpendicular to the plane of view.

Fig. 2: The sample is rotated by 90 degrees from (100) to (010) , yet the domain polarization seems to rotate from left to right, 180 degrees. This is another reason to better explain that polarization is angled into the image, along our field of view. Otherwise it is confusing for the reader.
Secondly: the domain structure in (b) and (c) seems consistent with a 45 degree rotation . But the domain structure in (a) seems very different. Is this problem with projection through overlapping domains along the field-of-view, or do the authors think the domain has re-arranged itself under the electron beam?

Lines 106-107 & 115-116: This is the measurement of the projected in-plane component of the polarization. Actual polarization is predominantly along <111>. Please be clear about this.

Figure S3. Needs a scalebar or peak-to-peak measurement included and preferably to have the profiles overlaid on each other for comparison.. It is difficult to see anything other than the presence of FFT spots from the figure.

Figure S4. I interpret it as the in-plane coefficient of polarization is reduced at the domain wall, and at the needle tip surface to avoid a charged surface. This makes sense theoretically, but is an interesting experimental observation, worth commenting on.

Figure S6 (a). the caption appears to be wrong. It is just a HAADF image, not a polarization map.

Lines 176-182, Figure S8, S9, : this is a good argument for why the domains point where they do. I would suggest making it more clear by including the polarization arrows in Supplementary figure S8, marking the arrow head as + and the tail as - . In a nanoneedle sample, with more surface strain relaxation available, the ferroelastic constraint on strict polarization directions is relaxed. This is seen in the bloch-like behaviour of the polarization maps in Figure 2. Hence domains are more free to react to local charging effects, so the presence of surface charge is a likely determining factor in local polarization and the stabilization of charged domain walls.

Figure 3b: since polarization is along <111> the out of plane lattice constant doesn’t correlate much with polarization. It may be more beneficial for the reader to see a map of the (110) spacings which might give an indication of ferroelastic effects.

Lines 212-214: These should refer back to figure S4 where there is experimental evidence of this phenomenon in the reduced in-plane coefficient of the polarization at the sample surface.

Line 422: Atomap and Temul software packages should have citations.

Kind Regards
Kalani Moore

Reviewer #3

(Remarks to the Author)
I co-reviewed this manuscript with one of the reviewers who provided the listed reports. This is part of the Nature Communications initiative to facilitate training in peer review and to provide appropriate recognition for Early Career Researchers who co-review manuscripts.

Version 1:

Reviewer comments:

Reviewer #1

(Remarks to the Author)
The manuscript has been improved compared to the previous version. The authors have also revised some of the claims in the manuscript through data analysis. I understand the difficulties in sample preparation. Although the revised manuscript is still based on limited samples, the authors' conclusions are logically reasonable based on the currently available data. As such, I agree to accept the manuscript after the following revisions are made:

1.In several figures within the maintext and the Supplementary Information, the crystallographic projections indicated in the captions or labels do not match the actual crystallographic directions shown in the images. Please carefully verify all figures against the correct sample orientation and revise the captions or labels accordingly.

2.The summary section of the Supplementary Information has not been updated to reflect the changes made to the figures and remains as the previous version. Please update this section accordingly.

Once these corrections are made, I recommend acceptance without further review.

Reviewer #2

(Remarks to the Author)
I am satisfied that my comments have bee addressed.
I am happy to recommend publication of the manuscript.

Kind regards
Kalani Moore

Version 2:

Reviewer comments:

Reviewer #1

(Remarks to the Author)
The labels in Figure S4 are a(100) b(100) c(010), but the captions are a (100) b(010) c(110).Please check your maintext and figures to ensure the consistency. Figure 2 has similar issue. Please check all your captions due to the figure changes in the revised version.

Once these corrections are made, I recommend acceptance without further review.

Open Access This Peer Review File is licensed under a Creative Commons Attribution 4.0 International License, which

permits use, sharing, adaptation, distribution and reproduction in any medium or format, as long as you give appropriate credit to the original author(s) and the source, provide a link to the Creative Commons license, and indicate if changes were made.
In cases where reviewers are anonymous, credit should be given to 'Anonymous Referee' and the source.
The images or other third party material in this Peer Review File are included in the article's Creative Commons license, unless indicated otherwise in a credit line to the material. If material is not included in the article's Creative Commons license and your intended use is not permitted by statutory regulation or exceeds the permitted use, you will need to obtain permission directly from the copyright holder.
To view a copy of this license, visit https://creativecommons.org/licenses/by/4.0/

RESPONSE TO REVIEWER COMMENTS – Submission NCOMMS-25-46220

Below we address all comments, suggestions and concerns raised by the three reviewers. For clarity, the referees' comments are bold. Changes most relevant to the reviewers comments are highlighted in yellow in the revised manuscript and referenced changes to the main figures are highlighted with red dashed lines.

Reviewer #1 (Remarks to the Author):

The authors observed charged domain wall and investigated the surface boundary in the ending of the $BiFeO_3$(BFO) nanoneedles by scanning transmission electron microscopy. The author also used phase-field simulations to investigate the formation mechanism of CDWs within BFO nanoneedles. While the manuscript explores an interesting topic in ferroelectric CDWs, the current version does not sufficiently demonstrate its scientific significance or provide robust data to support the conclusions. I will not recommend this paper for publication on Nature Communication unless the following major issues are addressed. Below are my specific comments.

Comment 1. The authors claimed that surface curvature plays a role in polarization domain control. The direction of the average measured polarization is within the quadrant with the highest curvature. This conclusion is insufficient unless multiple nanoneedles which have highest curvature point to different quadrants are measured. What's the initial polarization of the BFO film? Does the average polarization relevant to the initial polarization of the BFO film?

Response 1:

We would like to thank the reviewer for the useful suggestion and agree that the surface curvature analysis would significantly benefit from the inclusion of multiple nanoneedles. However, the precise control of the surface curvature is currently limited by the resolution of Ga ion-based FIB systems, particularly at low-kV milling. This restricts the success rate of fabricating nanoneedles with tip radius and surface curvature consistently comparable to that of the nanoneedle presented in the manuscript. We do however, present a surface curvature comparison between the nanoneedle surface with CDWs and the nanoneedle surface with a lower curvature magnitude and greater symmetry generated by Ar polishing of the surface using a Fischione nanomill presented in Fig. 3. It is demonstrated in this nanoneedle surface the reconfiguration of the polarization into a Neel type domain.

To refine the curvature analysis at the surface of the nanoneedle, we use the Frenet curvature formula to measure the varying curvature along the surface of the nanoneedle. We first implement this to the surface polynomial curvature analysis in supplemental figure S7 and Table S1. From the new calculations, a curvature asymmetry was observed, however it did not align to the net polarization direction as previously anticipated. It is likely that the polarization rotation is contingent not on the curvature magnitude but the strength of the curvature gradient. The calculation of the strength of the curvature gradient requires an

accurate position of the arc distances between surface atoms along the surface. However, some atomic positions cannot be determined with confidence from the integrated HAADF intensity profile and may render this quantification inaccurate for calculation of the curvature gradient along the needle surface. Therefore, we will omit this analysis from the supplemental information. Nonetheless, we will show in response to comment 2 that the Frenet equation can be applied to the main curvature analysis with a calculation of the curvature gradients.

To address the second part of comment 1, we included a new acquired HAADF-STEM cross section image of the original pristine thin film sample to figure 2 of the manuscript. Specifically, the HAADF image was captured 200nm from the BFO/TSO interface, that is the same distance as the nanoneedle tip to the interface. The polarization mapping of the as-grown sample in figure 1e below suggests the polarization in this region typically a monodomain without any charged domains residing at or around this region. The polarization observed in the bulk thin film is consistent with the polarization observed from the apex of the nanoneedle and suggests the average polarization of the fabricated nanoneedle is likely associated with the initial polarization of the pristine film.

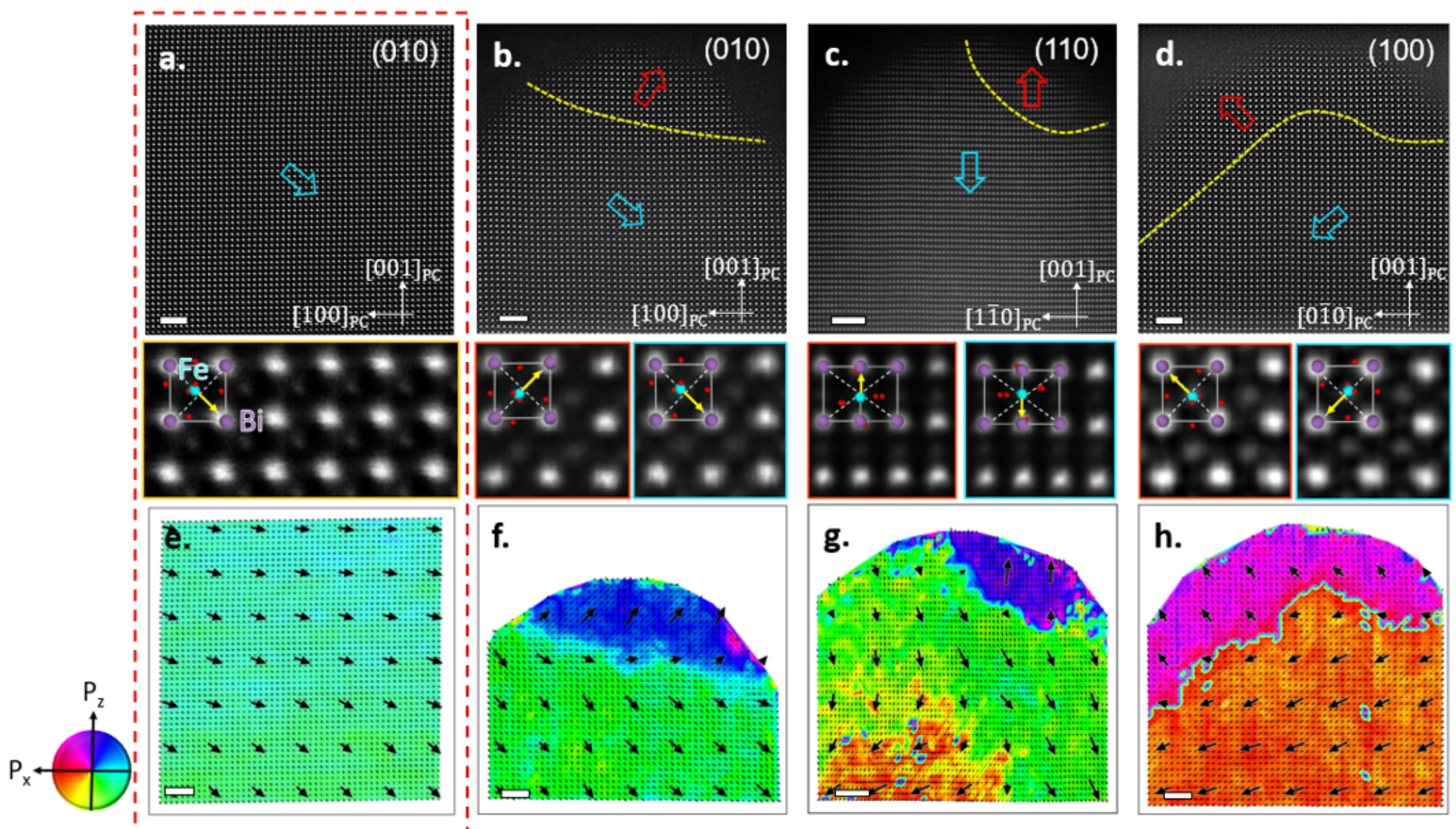

Figure 1: (a,b,c,d) HAADF-STEM images of the pristine BFO film, 200 nm from the BFO/TSO interface, and needle tip region of (100), (110) and (010) projections, respectively. The dashed yellow lines represent the domain wall between the up and down domain states represented by the red and cyan arrows. The up and down polarization states are highlighted in the HAADF sub-images. (e,f,g,h) Corresponding polarization vector mapping in the pristine and nanoneedle tip region. Scale bar is 2nm.

Comment 2. The authors clarified the CDW is stabilized. How to get a stabilized CDW? What's the critical condition? Under what kind of curvature? Or at what level of asymmetry? The paper currently lacks sufficient data on the manipulation of the CDW by surface curvature.

Response 2:

We appreciate the constructive suggestion. For the uniform needle surface as shown in Figure 3a and 3b, closer observation of the polarization configuration in uniform surface apex led us to characterize the polarization in this region as a Neel-type polarization. A similar polarization configuration has been observed in other BFO films also grown on a TSO substrate with charged domains[2]. Therefore, a critical curvature would allow us to see the transition between a Neel type and the Bloch-like configuration associated with the charged domain wall at the apex of the nanoneedle. Considering the simulated needle has a more Neel-type polarization near the apex, the CDW stability is likely dependent on the overall uniformity of the needle apex surface rather than just the magnitude of curvature (dT/ds). Therefore, we calculated the curvature gradients. We implemented the general parametric curvature formula (in the 2D case) to fit a B-spline along surface atomic positions, that can be observed as a parametric curve and quantify more accurate curvatures of our nanoneedle surface of the main text figures[1]. The results of this new curvature analysis for the needle with CDW are shown in Figure 2. Compared to the original curvature analysis in the submitted manuscript, the new curvature analysis along the (100) and (010) projections demonstrate localized high curvature "hotspots" along the surface. It is notable that the max curvature values for the apex with charged domains are 0.82 nm^{-1} and 0.87 nm^{-1} for the (100) and (010) projections, respectively, comparable to the max value 0.83 nm^{-1} found in simulation, while the max curvature in the needle with uniform polarization was only 0.59 nm^{-1}.

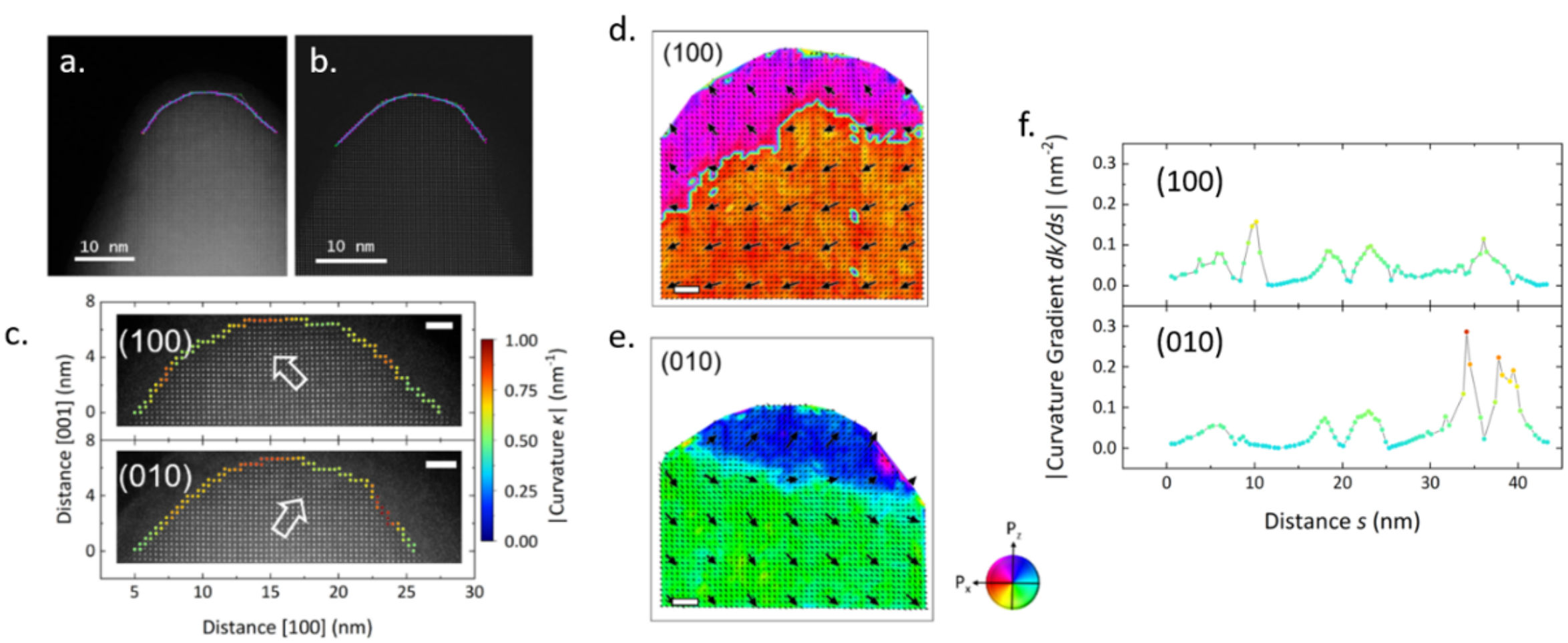

Figure 2: Non-uniform surface with Charged Domain (a,b) Parametric fitting line for curvature analysis for (100) and (010) projections. (c) The measured surfaces curvatures at the apex surface of the BFO needle along the (100) and (010) projections. (d,e) Polarization mapping at the (100) and (010) projections demonstrating a charged domain wall. (f) Curvature gradients for the (100) and (010) projections. Dashed line represents the approximated critical curvature gradient for charged domain formation. Scalebar = 2nm

From the calculated curvature gradient in figure 2f, the position and relative magnitude of the curvature hotspots were determined such that the polarization at the needle tip is orientated toward the surface of overall highest curvature gradient. Therefore, we claim that an asymmetric surface could rotate polarization to high curvature gradient surfaces to compensate for the surface charges. To quantify the critical surface curvature gradient, the same curvature analysis was performed on the nanoneedle surface exposed to Ar polishing as shown in figure 3d. The curvature gradient maximum magnitude is 0.05 nm^{-2} in this nanoneedle surface. In the nanoneedle surface with CDWs, the minimum curvature gradient magnitude where polarization rotation was observed is 0.07 nm^{-2}. Therefore, we estimate the critical curvature gradient to be near 0.07 nm^{-2}.

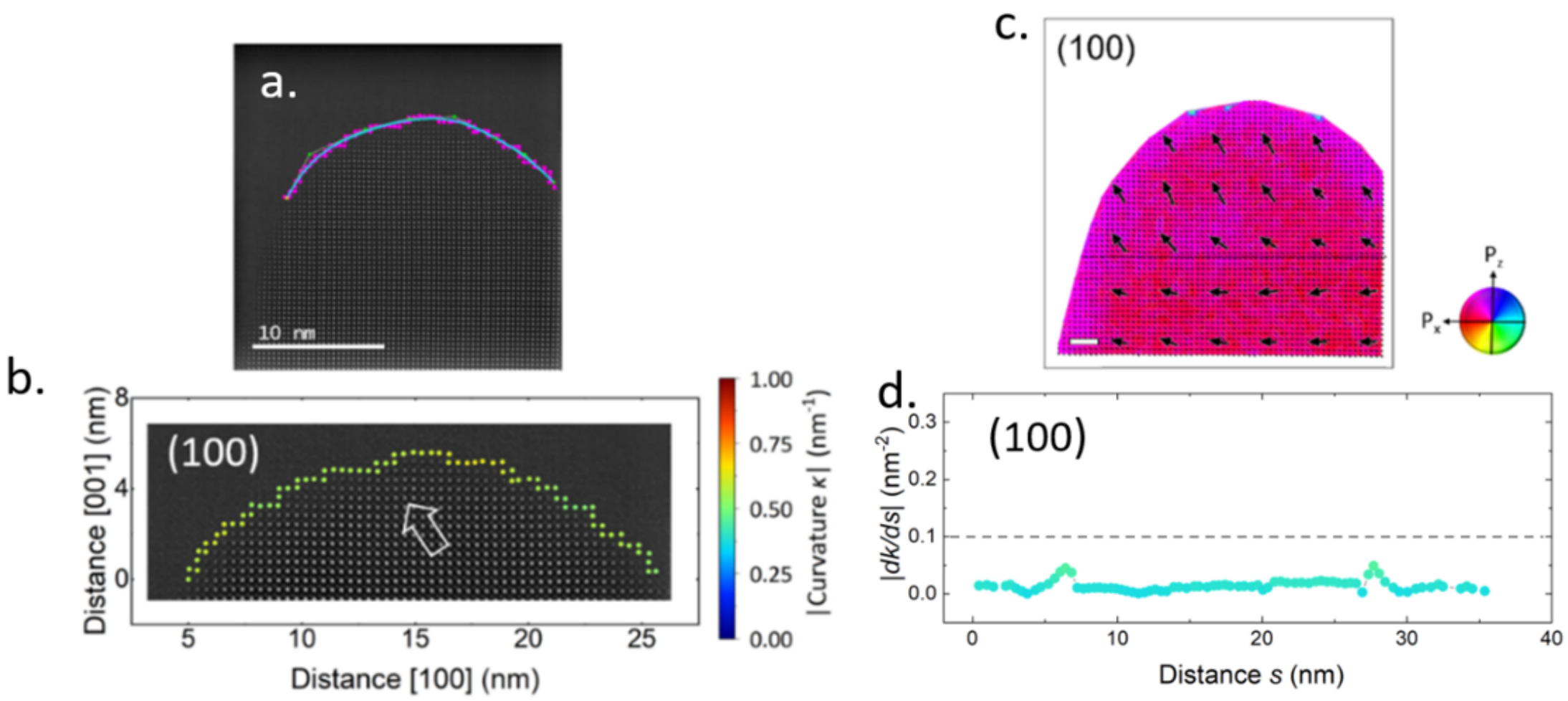

Figure 3: Uniform Surface with Partial Flux Closure (a) Parametric fitting line for curvature analysis for (100) projections with uniform surface (b) The corresponding measured surface curvatures at the apex surface of the BFO needle. (c) Polarization mapping demonstrating a partial flux closure domain. (d) Corresponding curvature gradient plot of figure b, demonstrating a relatively uniform curvature at the nanoneedle surface. Scalebar = 2nm

Comment 3. Can the stabilized CDW be read out or manipulated by electric field? What's the application prospects of this stabilized CDW in the specific nanoneedle structure?

Response 3:

We appreciate this useful comment on manipulation of CDWs in BFO nanoneedle structures. Considering that this experiment on nature of ferroelectric domains in nanoneedles is a first of its kind, more of the application prospects will be explored in further studies. Nonetheless, we expect that the CDWs should be able to be read out in principle, however movement of the CDW may be limited due to strain gradient pinning and the geometrical constraints near the apex. However, geometric pinning may offer a very

stable CDW that can be read out and switched by means of applied electric field with high repeatability. The application prospects may involve nanoscale memory devices based on geometry defined architectures where an array of curvature controlled nanoneedles be used to create reliable high-density memory devices. However, this study is fundamental in nature and offers insight that could guide future studies based on nano-fabrication, surface manipulation and defect engineering in ferroelectric devices.

Comment 4. The titles of the paper should be unified. Currently, the titles of the main text and the SI are not consistent.

Response:

We have corrected the title mistake in the supplemental information document.

Reviewer #2 (Remarks to the Author):

Overall I find this work to be of significant merit and contains the noteworthy observation of charged tail-to-tail domain walls at the tip of a BiFeO3 nanoneedle. The authors performed detailed experimental work using STEM imaging and 4D-STEM at the atomic level to provide evidence for the reasons for the domain structure formation. Polarization mapping from the STEM imaging and charge density observations from 4D-STEM are excellent tools to gain a clear understanding of the ferroelectric domain structure. Phase-field modelling supported their conclusions. There has been previous work using FIB milling showing how nanoscale geometric confinement affects ferroelectric domain structures, (e.g. Schilling, A., et al. "Morphological control of polar orientation in single-crystal ferroelectric nanowires." Nano letters 7.12 (2007): 3787-3791.) and charged domain walls in similar doped materials have been reported (e.g. Moore, Kalani, et al. "Charged domain wall and polar vortex topologies in a room-temperature magnetoelectric multiferroic thin film." ACS Applied Materials & Interfaces 14.4 (2022): 5525-5536.). However, those did not use pure $BiFeO_3$ and did not use nanoneedle geometry. As mentioned by the authors, the sample-substrate geometry is critical in controlling strain relaxation to govern specific domain pattern formation. The insight in this work that surface angle, and the specific associated surface charge effects can induce charged domain wall formation is notable. The experimental evidence is thorough, and though the number of observations/samples analyzed is small, the conclusions are logical and well-reasoned. There is certainly enough detail provided for the community to reproduce and build on the work.

Below are specific comments that I believe will improve the interpretation and clarity of the work for the reader.

Line 48 : This should cite ‘H. Lu et al. Electrical Tunability of Domain Wall Conductivity in LiNbO3 Thin Films. Adv. Mater. 2019, 31, 1902890. https://doi.org/10.1002/adma.201902890’

Response:

We have fixed this citation error.

Lines 54-56: This comment about domain wall surface curvature extrapolated from a different material system isn't well justified or very helpful. Domain wall surface curvature is generally energetically unfavorable. Indeed this is why most bulk domain walls are very straight. Exploring the situations where domain walls are curved is very interesting, but that is a consequence of the sample surface geometry that forces the domain walls to adapt. This is explained well in the discussion, and would be better context to provide in the introduction.

Response:

We thank the reviewer for this insightful comment. To this end, we have added some comments to the introduction section regarding the stability of ferroelectric domains in response to the depolarization field. We also added a reference that offers an example where depolarization fields drive a metastable ferroelectric state in a HZO film. This acts as a prelude toward how our modified surface boundary conditions responses to the geometrically enhanced depolarization field leading to the stable charged domains observed in our BFO nanoneedle.

Lines 61-62: This makes it sound like the domain structures are needles. Better to say Needle shaped samples. The most common use of needle-samples fabricated by FIB is for Atom Probe tomography. This should probably be mentioned.

Response:

We thank you for the suggestion. We have added the suggested comments to lines 61-62.

Fig1 c, d: Should attempt to convey that the allowed bulk polarization direction in BFO are from the Fe, angled towards a corner Bi atom along <111>. Not parallel/perpendicular to the plane of view.

Response:

We appreciate the suggestion to include the 3-dimensional polarization <111>. To this end, we have added a 3D crystal structure with a <111> vector representing the direction of polarization in a BFO unit cell.

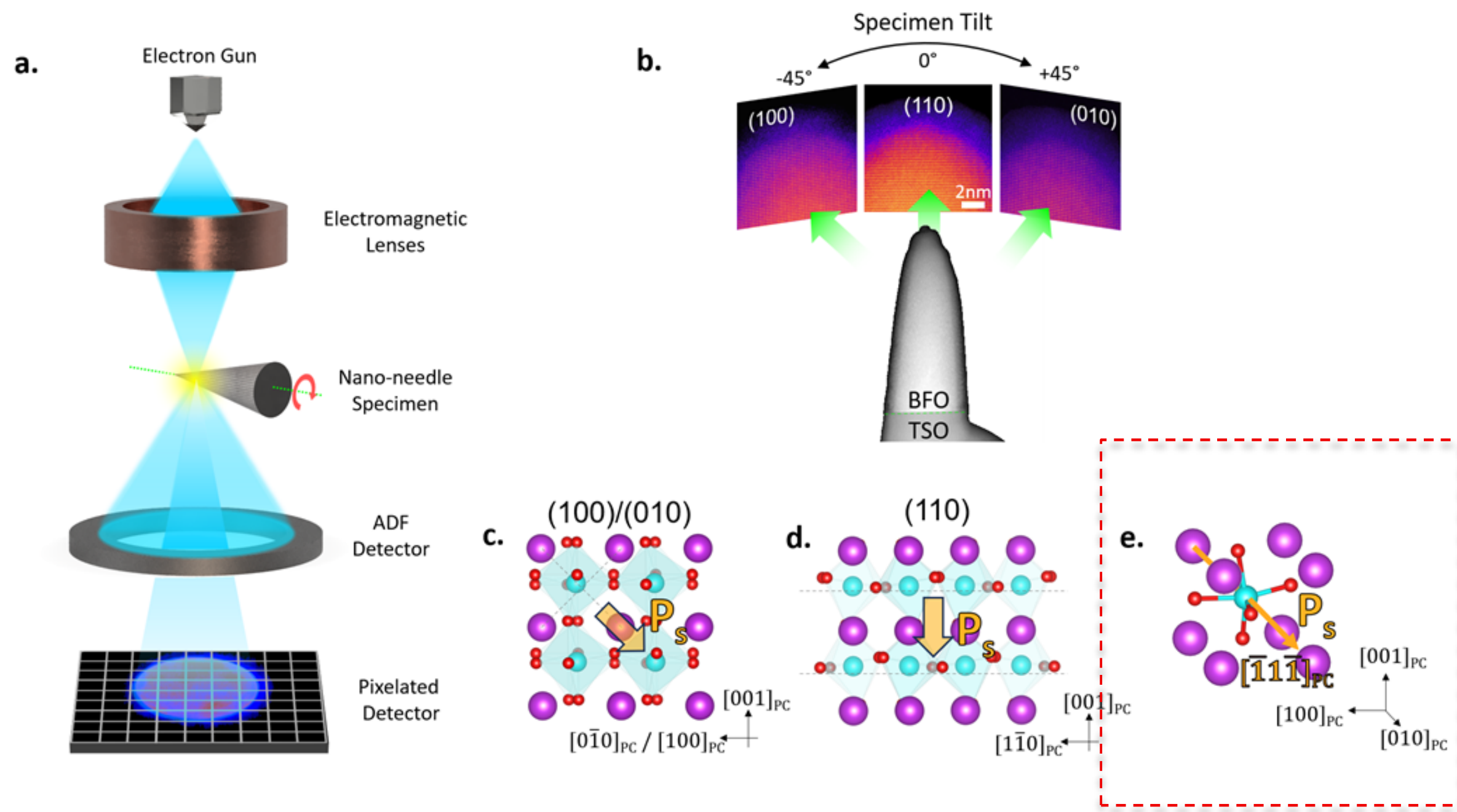

Figure 1 | High-symmetry Tilt Projection Series on $BiFeO_3$ nanoneedle. (a) Schematic diagram of scanning electron microscope. Needle sample are tilted around a tilt axis to obtain low order tilt projections. ADF detector and pixelated detector collect HAADF and 4D-STEM CBED patterns, respectively. (b) STEM tilt series of high-symmetry projections at needle tip. (c,d) BFO crystal structural model for pseudo-cubic high-symmetry tilt projection series of the (100) and (010) projections in figure (c) and (110) projection in figure d. (e) 3D BFO pseudocubic unit cell structure with 3D polarization vector in the $[111]_{PC}$ direction.

Fig. 2: The sample is rotated by 90 degrees from (100) to (010) , yet the domain polarization seems to rotate from left to right, 180 degrees. This is another reason to better explain that polarization is angled into the image, along our field of view. Otherwise it is confusing for the reader.
Secondly: the domain structure in (b) and (c) seems consistent with a 45 degree rotation . But the domain structure in (a) seems very different. Is this problem with projection through overlapping domains along the field-of-view, or do the authors think the domain has re-arranged itself under the electron beam?

Response:

Thank you for your comment. Regarding the differences between figure (a) and figures (b) and (c) we have made a connection between surface curvature gradient and the formation and size of the charged

domain near the apex of the nanoneedles. It seems that the distribution of high curvature gradients along the surface can affect the shape of the charged domain wall. Therefore, this may explain why the domain structure in (a) appears very different from the domain structures of (b) and (c) This is explained in greater detail in the response to comment 2 from reviewer #1.

Lines 106-107 & 115-116: This is the measurement of the projected in-plane component of the polarization. Actual polarization is predominantly along <111>. Please be clear about this.

Response:

We thank the reviewer for the comment of providing additional clarification of the 3D polarization of BFO. We have added a statement to clarify this following the statement of the determination of the polarization using 2D images. This should help the reader distinguish between the polarization observed in 2D images vs the real polarization in 3D.

Figure S3. Needs a scalebar or peak-to-peak measurement included and preferably to have the profiles overlaid on each other for comparison. It is difficult to see anything other than the presence of FFT spots from the figure.

Response:

We thank the reviewer for their comment. We have modified supplemental figure 3 (S3) to include more FFT patterns with their corresponding profiles. We measured the distance between FFT spots individually at each region for comparison. The measured lattice spacing at the three regions suggests that the BFO structure is preserved throughout the entire nanoneedle sample.

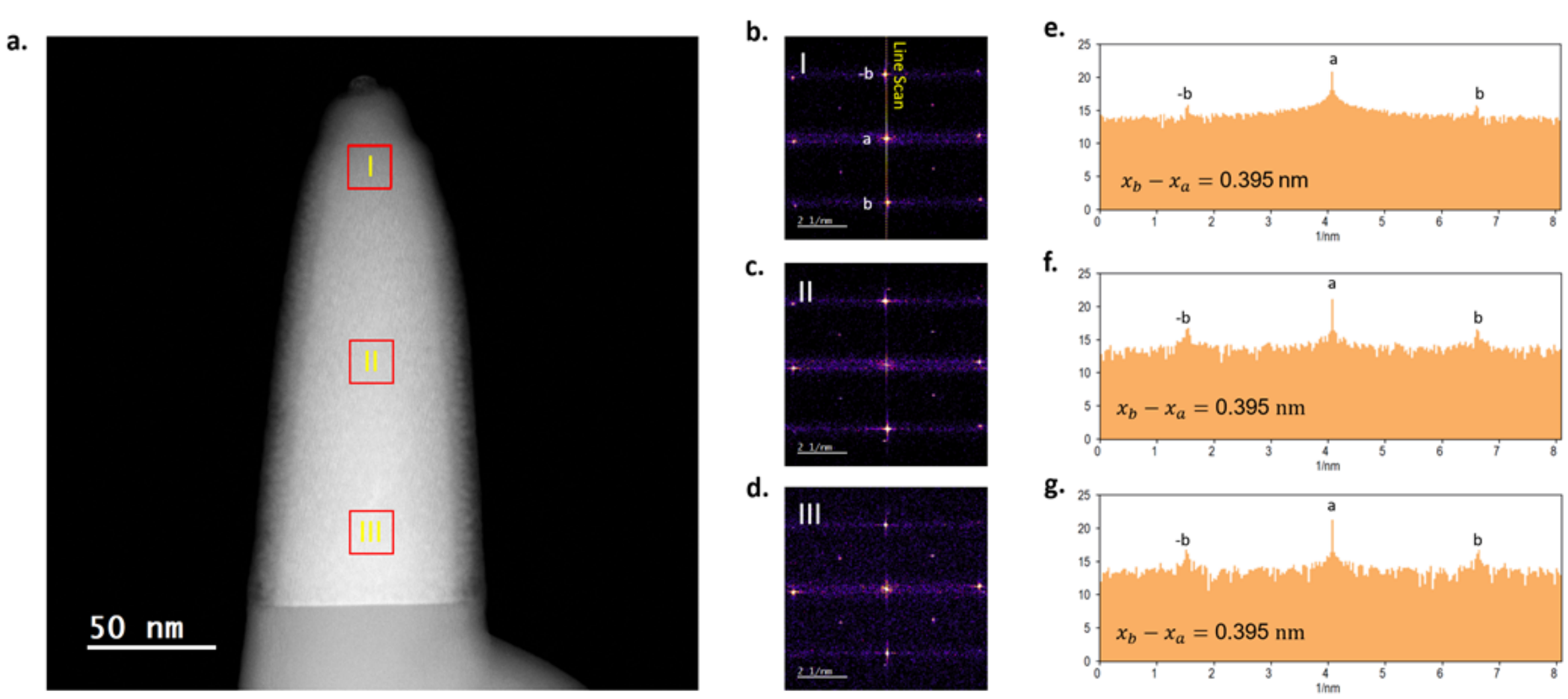

Figure S3 | BFO Lattice Parameter. (a) Post annular milled BFO nanoneedle specimen. Red boxes represent regions of acquired FFT. (b,c,d) FFTs acquired at regions I,II and III in figure a. (e,f,g) FFT line profiles across FFT peaks along dashed line in figure b for regions I,II and III respectively.

Figure S4. I interpret it as the in-plane coefficient of polarization is reduced at the domain wall, and at the needle tip surface to avoid a charged surface. This makes sense theoretically, but is an interesting experimental observation, worth commenting on.

Response:

We appreciate the reviewer's insight that helped to greatly improve our manuscript. As mentioned, we associated the reduction in the in-plane coefficient as evidence toward a Bloch-like polarization at the CDW. We then compared this with the polarization at the modified needle surface (smooth) that revealed a Neel-type polarization. With this information we determined that a greater surface curvature gradient may lead to a Neel to Bloch-like rotation, where Bloch type is typically associated with Ferroelastic effects, and therefore help to explain why the charged domain walls are stabilized at the nanoneedle apex.

However, we didn't previously consider the reduced in-plane polarization near the surface. New projected Fe charge maps that were generated from the charge density maps were added to main figure 3. The projected charge along the (100) projection demonstrates a reduced charge at the surface that may support the reduction in the in-plane polarization. The enhanced polarization in the

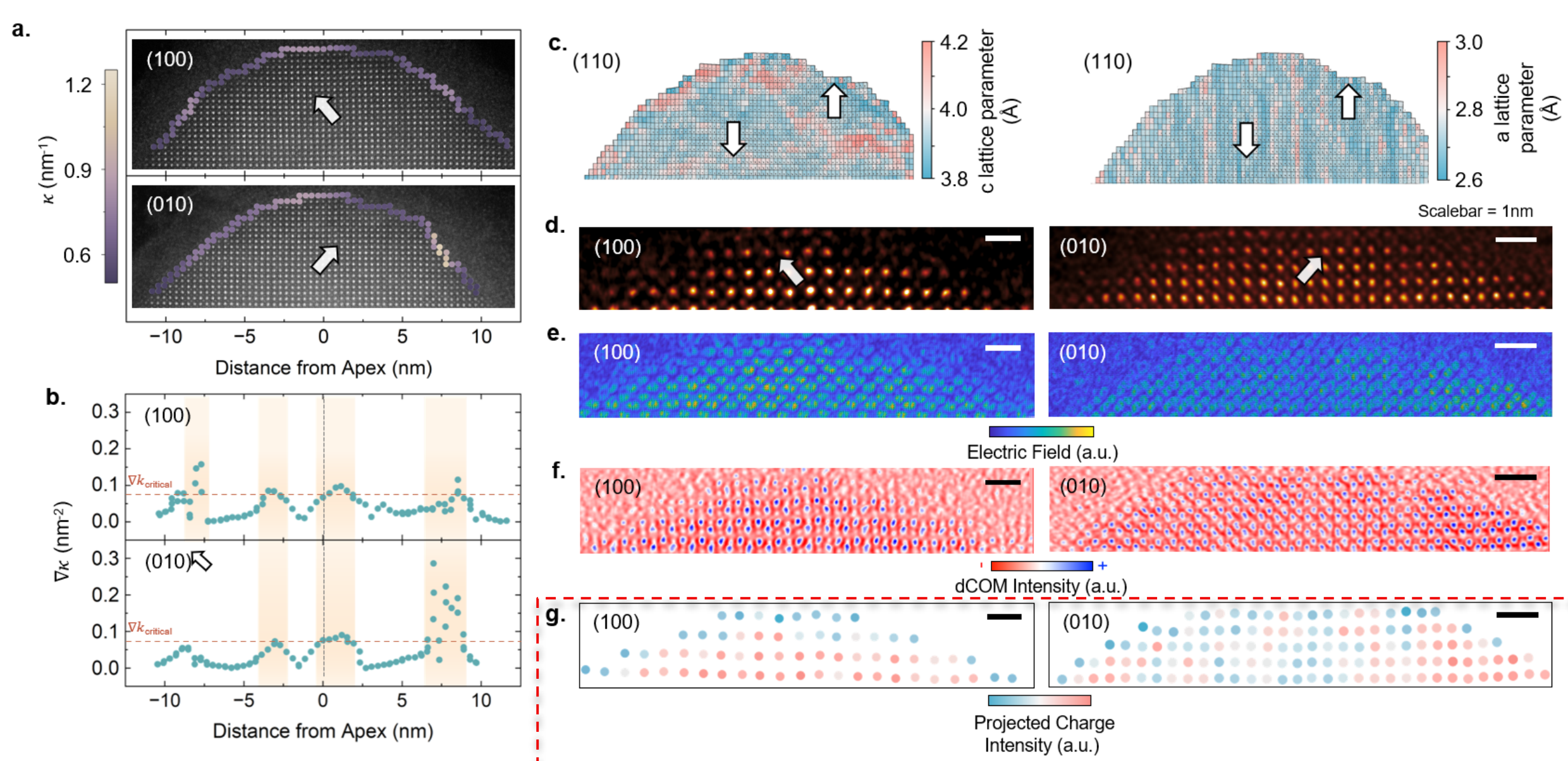

Figure 3 | Surface Analysis. (a) Curvature of surface of (100) and (010) projections derived from B-spline fitting and curvature calculation using the general curvature formula. (b) Corresponding curvature gradient plots of figure a,

demonstrating variations in surface curvature at the nanoneedle surface. Dashed line represents the approximated critical curvature gradient for charged domain formation. (c) Unit cell resolved out-of-plane normal strain at BFO nano needle surface of (110) projection. A ferroelastic gradient resides at the domain wall. (d) Simultaneously captured ADF image for (100) projection at needle tip. (e) Map of the reconstructed electric field distribution in the same region as figure (c). (f) Projected electron charge density map calculated from electric field in (b). (g) Integrated charge intensity at cation atomic sites using Voronoi integration technique. The purple and cyan circles are the Bi and Fe termination sites. Scale bar is 2nm.

Figure S6 (a). the caption appears to be wrong. It is just a HAADF image, not a polarization map.

Response: We have fixed this mistake.

Lines 176-182, Figure S8, S9, : this is a good argument for why the domains point where they do. I would suggest making it more clear by including the polarization arrows in Supplementary figure S8, marking the arrow head as + and the tail as - . In a nanoneedle sample, with more surface strain relaxation available, the ferroelastic constraint on strict polarization directions is relaxed. This is seen in the bloch-like behaviour of the polarization maps in Figure 2. Hence domains are more free to react to local charging effects, so the presence of surface charge is a likely determining factor in local polarization and the stabilization of charged domain walls.

Response:

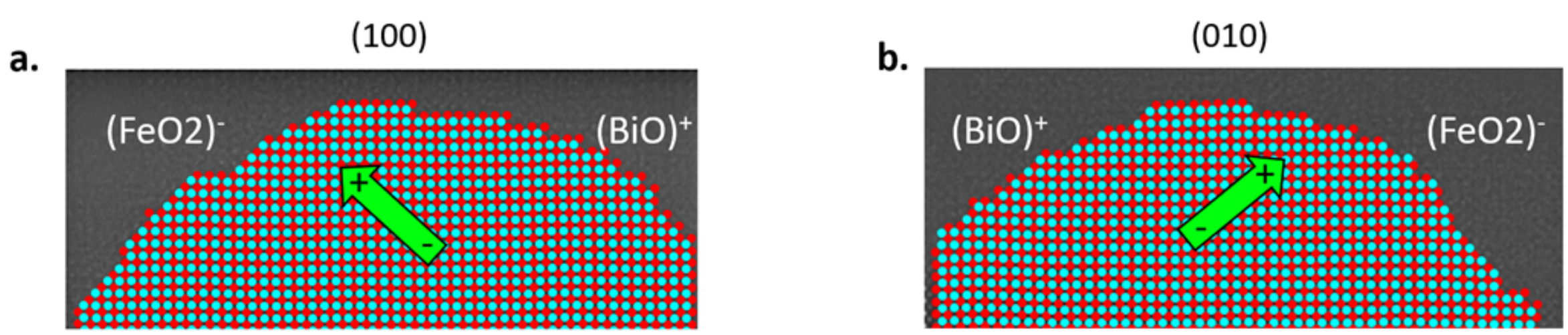

Figure S8 | Surface Terminations. (a,b) 2D-Gaussian fitted Bi and Fe atomic positions for the (100) and (010) HAADF projections at the BFO needle tip region. Higher surface curvatures expose Fe atoms on the surface leading to different terminations across the needle surface. Bi and Fe atoms are represented by the red and cyan circles, respectively.

Figure 3b: since polarization is along <111> the out of plane lattice constant doesn't correlate much with polarization. It may be more beneficial for the reader to see a map of the (110) spacings which might give an indication of ferroelastic effects.

Response:

We thank the reviewer for the suggestion to recommend the (110) lattice spacing map instead of the (100) spacing map. We have included (110) spacing maps of the c and a lattice parameter to the main Figure 3. Between the c and a lattice spacing, the c lattice spacing is noted to partake in the flexoelectric elastic effect at the CDW. This has strengthened our discussion surrounding the strain effects toward the stability of CDWs in our nanoneedle.

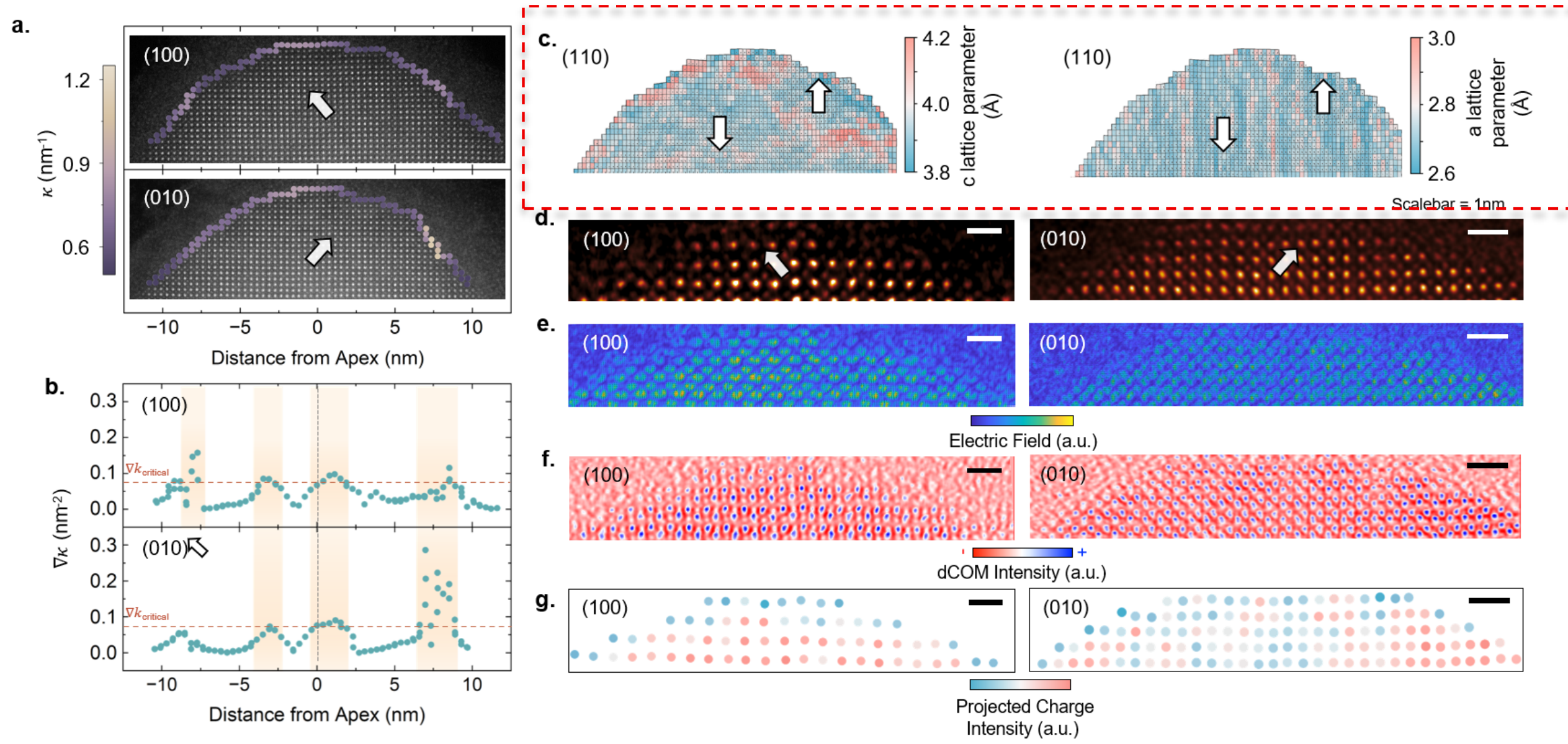

Figure 3 | Surface Analysis. (a) Curvature of surface of (100) and (010) projections derived from B-spline fitting and curvature calculation using the general curvature formula. (b) Corresponding curvature gradient plots of figure a, demonstrating variations in surface curvature at the nanoneedle surface. Dashed line represents the approximated critical curvature gradient for charged domain formation. (c) Unit cell resolved out-of-plane normal strain at BFO nano needle surface of (110) projection. A ferroelastic gradient resides at the domain wall. (d) Simultaneously captured ADF image for (100) projection at needle tip. (e) Map of the reconstructed electric field distribution in the same region as figure (c). (f) Projected electron charge density map calculated from electric field in (b). (g) Integrated charge intensity at cation atomic sites using Voronoi integration technique. The purple and cyan circles are the Bi and Fe termination sites. Scale bar is 2nm.

Lines 212-214: These should refer back to figure S4 where there is experimental evidence of this phenomenon in the reduced in-plane coefficient of the polarization at the sample surface.

Response:

We thank the reviewer for this useful suggestion. We have added a statement to the main text that refers to the experimentally observed reduction of the in-plane coefficient of the polarization shown in figure S4 to the observed arrangement of the phase-field simulated polarization that reduces the depolarization field.

Line 422: Atomap and Temul software packages should have citations.

Response:

We have included the citations for the Atomap and Temul software packages in the reference section.

Kind Regards
Kalani Moore

Reviewer #3 (Remarks to the Author):

I co-reviewed this manuscript with one of the reviewers who provided the listed reports. This is part of the Nature Communications initiative to facilitate training in peer review and to provide appropriate recognition for Early Career Researchers who co-review manuscripts.

References:

[1] Hadrien M. and Brouhard G., Kappa (κ): Analysis of Curvature in Biological Image Data using B-splines, *bioRxiv* 852772 (2019).

[2] Linze L, Atomic Scale Structure Changes Induced by Charged Domain Walls in Ferroelectric Materials, *Nano Letters* **13** 5218-5223 (2013)

[3] Johnson C.A, Generalization of the Gibbs-Thomson Equation, *Surface Science* **3** 429-444 (1965)

RESPONSE TO REVIEWER COMMENTS – Submission NCOMMS-25-46220B

Reviewer #1 (Remarks to the Author):

The manuscript has been improved compared to the previous version. The authors have also revised some of the claims in the manuscript through data analysis. I understand the difficulties in sample preparation. Although the revised manuscript is still based on limited samples, the authors' conclusions are logically reasonable based on the currently available data. As such, I agree to accept the manuscript after the following revisions are made:

1.In several figures within the maintext and the Supplementary Information, the crystallographic projections indicated in the captions or labels do not match the actual crystallographic directions shown in the images. Please carefully verify all figures against the correct sample orientation and revise the captions or labels accordingly.

Response:

We thank the reviewer for bringing this to our attention. The crystallographic labeling has been revised throughout the main text figures and supplementary information to ensure consistency among the projections within a common pseudo-cubic coordinate system. In addition, Figure 1 has been updated to present the (100) and (010) projected crystal structures in separate figures, improving clarity in distinguishing the polarization direction along the three high-symmetry zone-axis projections ((100), (110) and (010)) and the corresponding crystal structure.

2.The summary section of the Supplementary Information has not been updated to reflect the changes made to the figures and remains as the previous version. Please update this section accordingly.

Response:

We have updated the summary section of the supplementary information file to reflect the new changes made to the figures.

Once these corrections are made, I recommend acceptance without further review.

Reviewer #2 (Remarks to the Author):

I am satisfied that my comments have been addressed.
I am happy to recommend publication of the manuscript.

Kind regards
Kalani Moore

RESPONSE TO REVIEWER COMMENTS – Submission NCOMMS-25-46220

Reviewer #1 (Remarks to the Author):

The labels in Figure S4 are a(100) b(100) c(010), but the captions are a (100) b(010) c(110).Please check your main text and figures to ensure the consistency. Figure 2 has similar issue. Please check all your captions due to the figure changes in the revised version.

Once these corrections are made, I recommend acceptance without further review.

Response:
We appreciate the reviewer for bringing this inconsistency to our attention. We have updated the captions of supplementary figure 4 and main figure 2 to fix the incorrect caption statements and make it consistent with their corresponding revised figures.
